# The tubulin inhibitor MG-2477 induces autophagy-regulated cell death, ROS accumulation and activation of FOXO3 in neuroblastoma

**DOI:** 10.18632/oncotarget.16434

**Published:** 2017-03-22

**Authors:** Judith Hagenbuchner, Lorena Lungkofler, Ursula Kiechl-Kohlendorfer, Giampietro Viola, Maria Grazia Ferlin, Michael J. Ausserlechner, Petra Obexer

**Affiliations:** ^1^ Department of Pediatrics II, Medical University Innsbruck, Innsbruck, Austria; ^2^ Department of Pediatrics I, Medical University Innsbruck, Innsbruck, Austria; ^3^ Tyrolean Cancer Research Institute, Innsbruck, Austria; ^4^ Department of Woman's and Child's Health, Oncohematology Laboratory University of Padova, Padova, Italy; ^5^ Department of Pharmaceutical and Pharmacological Sciences, University of Padova, Padova, Italy

**Keywords:** tubulin-inhibition, autophagy, NOXA, BCLXL, BIRC5/Survivin

## Abstract

Neuroblastoma is the most frequent extra-cranial solid tumor in children with still high mortality in stage M. Here we studied the tubulin-inhibitor MG-2477 as a possible therapeutic agent for neuroblastoma therapy and uncovered that MG-2477 induces death in neuroblastoma cells independent of PKB-activation status and stage. MG-2477 triggers within 30 minutes extensive autophagosome-formation that finally leads to cell death associated with mitotic catastrophe. Autophagy is critical for MG-2477-induced death and is regulated by the BH3-only protein PMAIP1/NOXA which sequesters the anti-apoptotic BCL2-protein BCLXL and thereby displaces and activates the autophagy-regulator BECN1/beclin1. Knockdown of NOXA or overexpression of its pro-survival binding partners MCL1 and BCLXL counteracts MG-2477-induced cell death. MG-2477 also rapidly induces the repression of the anti-apoptotic protein Survivin, which promotes autophagy and cell death. We further observed the accumulation of reactive oxygen species (ROS) that triggers autophagy induction suggesting a change of the PI3 kinase-III/BECN1 complex and activates the transcription factor FOXO3, which contributes to final cell death induction. The combined data suggest that MG-2477 induces a sequential process of ROS-accumulation, autophagy and FOXO3-activation that leads to cell death in neuroblastoma cells.

## INTRODUCTION

Neuroblastoma is a pediatric malignancy that develops from undifferentiated precursors of the sympathetic nervous system. Depending on stage, patients with neuroblastoma face a poor prognosis and survival rate, especially children older than one year with metastatic stage M (old classification stage IV) tumors [1]. Due to the broad range of genetic alterations, like genomic amplification of NMYC, the gain of chromosome 17q or hyperactivation of pro-survival phosphatidylinositol-3-kinase (PI3K)/protein kinase B (PKB) signaling, high stage neuroblastoma tumors poorly respond to radiation therapy and are frequently resistant to chemotherapeutic agents [2–4]. Especially the gain of chromosome 17q leading to enhanced BIRC5/Survivin expression [3] contributes to resistance against DNA-damaging agents, since Survivin efficiently prevents reactive oxygen species (ROS) signaling and the further activation of downstream death signaling cascades [5, 6].

For many years, anti-mitotic drugs have been used for treating a variety of malignancies. These compounds are frequently of natural origin and possess high anti-proliferative effects [7]. In principal, there are two major classes of compounds which interfere with microtubule dynamics: Inhibitors of the tubulin assembly into microtubule structures or the inhibitors of microtubule disassembly. In principal, they can bind to three different sites of tubulin, the paclitaxel site, the vinca-domain or into the colchicine site [8]. Both classes inhibit mitosis and induce therefore either mitotic cell death or cell death in G1-phase after slippage of tetraploid/aneuploid daughter cells into the cell cycle [9]. Since cancer cells are fast dividing cells, improvement of these substances is still of high scientific interest to overcome resistance. Moreover understanding their modes of action might also improve combination therapies and reduce effective doses. Anti-mitotic drugs possess high cytotoxic potential, since they target a large number of signaling pathways and proteins and many reports suggest the involvement of autophagy related genes as well as members of the BCL2 family [10, 11].

Programmed cell death can be divided into four major forms: in type I – apoptosis, which is induced either by extrinsic or intrinsic signaling pathways that converge on caspase activation and DNA fragmentation [12], in type II – autophagic cell death, in mitotic catastrophe and in regulated necrosis [13].

In most cases cell death execution is not the result of single pathway activation, but the consequence of cross-talks between different cell death signaling pathways and pro-survival signals. The interplay of these pathways is complex since they can be activated simultaneously and frequently the involved key players participate in more than one signaling cascade. This especially includes the family of BCL2 proteins, which is critically involved in the regulation of all forms of cell death. The BCL2 protein family is composed of pro-apoptotic BH3-only proteins, such as BCL2L11/BIM, NOXA and the autophagy regulator BECN1/beclin1 [14], pro-apoptotic BCL2 proteins of the BAX-BAK family that control outer mitochondrial membrane permeabilization and the pro-survival BCL2 partners BCL2, BCL2L1/BCLXL, BCL2L2/BCLW, MCL1 and BCL2A1. Different models on how these proteins interact with each other and steer death decision have been proposed [15]. Importantly, pro-survival proteins such as BCL2 and BCLXL, but also pro-apoptotic BIM interact with BECN1 and other autophagy regulators and thereby directly modulate autophagy induction [16, 17]. Since many stimuli trigger apoptosis and autophagy, autophagy is often observed before apoptosis induction as a rescue mechanism of the cell to adapt to stress [18]. In this case, however, autophagy inhibits apoptosis execution [19]. On the other hand, apoptosis can also inhibit autophagy by cleavage of autophagy-related genes like ATG3, BECN1, or AMBRA1 [14, 20–22]. The existence of autophagic cell death, a form of cell death mediated by autophagy in mammals is still under debate, since it does not have a role in mammalian development, but it was shown to contribute to drug-induced cell death execution in some cancer cells, especially in those lacking BAX and BAK1 or CASP3/Caspase 3 [23, 24]. Autophagy may facilitate apoptosis by degradation of inhibitory proteins, like IAPs (*I*nhibitor of *A*poptosis *P*roteins) [25] or even induce necrosis by degradation of enzymes involved in stress detoxification [26].

The tubulin-inhibitor 3-cyclopropylmethyl-7-phenyl-3*H*-pyrrolo[3,2-*f*]quinolin-9(6H)-one also named MG-2477 was obtained as described in Gasparotto et. al. [27]. MG-2477, which shares the three-ring system with flavones and colchicines, binds with high affinity to the colchicine site of tubulin and is cytotoxic to different cancer cell lines like non-small cell lung carcinoma, ovarian or breast cancer [11, 27]. Although there are some reports which demonstrate that anti-mitotic drugs are ineffective in cancers with increased/activated PKB levels [28–30], Viola and colleagues demonstrated that MG-2477 treatment de-phosphorylates and inactivates PKB [11]. Since this pathway is hyperactivated in human neuroblastoma [4], we investigated the potential cytotoxic effect of MG-2477 in different neuroblastoma cell lines [31]. The project was designed to study the initial phase of MG-2477-induced cell death and to investigate whether this drug may be a potential agent for the treatment of neuroblastoma. MG-2477 efficiently induced cell death in neuroblastoma cells independent of the PKB status, but did not affect differentiated neuronal cells. Upon addition of MG-2477 we observed rapid autophagy associated with accumulation of ROS, induction of NOXA and repression of Survivin. This initial phase was followed by activation of the transcription factor FOXO3 and cell death associated with signs of mitotic catastrophe and slippage into apoptosis.

## RESULTS

### MG-2477 triggers cell death in neuroblastoma cells independent of the PKB status

**Figure 1:**
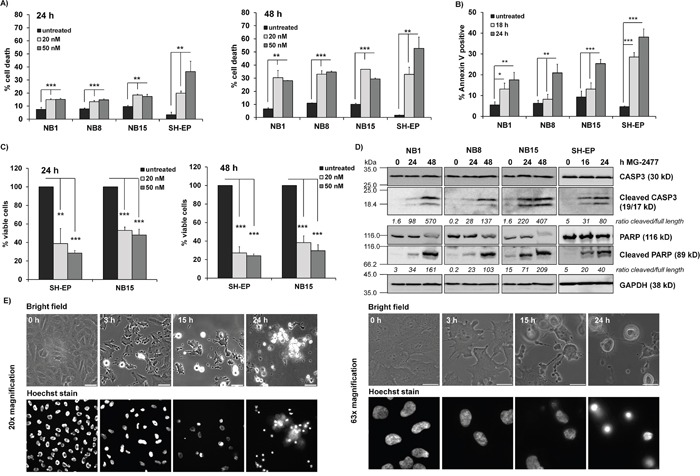
MG-2477 induces cell death in different neuroblastoma cell lines **(A)** The cell lines NB1, NB8, NB15, and SH-EP were treated for 24 (left panel) and 48 hours (right panel) with 20 and 50 nM MG-2477 or **(B)** subjected to Annexin V staining after 18 and 24 hours of treatment with 50 nM MG-2477. The cells were analyzed by flow cytometry **(A)** for PI staining of fragmented nuclei (three independent experiments) and **(B)** Annexin V positivity (four independent experiments). **(C)** Cell viability was assessed by AlamarBlue assay of SH-EP and NB15 cells after treatment with 20 or 50 nM MG-2477 for 24 (left panel) or 48 hours (right panel). Shown is the mean of five independent experiments. Statistical difference between untreated and MG2477-treated cells was assessed by unpaired t-test (significantly different: *P< 0.05; **P<0.01; ***P<0.001). **(D)** NB1, NB8, and NB15 cells were treated with 50 nM MG-2477 for 24 and 48 hours and SH-EP cells for 16 and 24 hours. CASP3 and PARP levels/cleavage were analyzed by immunoblot. GAPDH served as loading control. Densitometric analyses were done using Labworks software and expressed as cleaved/full length ratio. **(E)** SH-EP cells were treated with 50 nM MG-2477 up to 24 hours. Cell morphology was monitored by bright field microscopy after 3, 15 and 24 hours at 20x (left) and 63x (right) magnification; nuclei were stained with 100 ng/ml Hoechst33342 stain. Size marker in the left panel is 50 μm, in the right panel 20 μm.

MG-2477 was originally developed as an anti-mitotic drug suggesting that it affects mitotic spindle formation in neuroblastoma. As expected from its ability to bind to the colchicine site of tubulin, MG-2477-treated cells failed to organize polymerized microtubule network and further chromosome orientation and segregation (Supplementary Figure 1A). To investigate the effect of MG-2477 on deregulated PKB survival signaling, we chose a set of human neuroblastoma cell lines which vary in the expression and activity of PKB (Supplementary Figure 1B). The cell lines NB1, NB8, NB15 and SH-EP were treated with 20 and 50 nM MG-2477 for 24 and 48 hours. All tested cell lines showed increasing amounts of cells in sub-G1 already after 24 hours and reached a maximum of 30 to 50% death after 48 hours (Figure 1A). Additionally, MG-2477 treatment caused Annexin V positivity within 18 to 24 hours (Figure 1B) and was associated with CASP3 and PARP cleavage in all tested cell lines (Figure 1D) suggesting that a significant part of cells underwent apoptotic cell death. To exclude cytotoxic effects of MG-2477 on differentiated neuronal cells, we differentiated NB8 and NB15 cells with all-trans retinoic acid (RA) and pheochromocytoma PC12 cells with nerve growth factor (NGF) and treated undifferentiated as well as differentiated cells with increasing amounts of MG-2477. PC12 cells are derived from rat adrenal medulla and represent an established model for neuronal differentiation [32, 33]. Differentiated NB8, NB15, and PC12 cells remained resistant to MG-2477 up to a concentration of 100 nM, whereas undifferentiated cancer cells underwent significant cell death (Supplementary Figure 2). This suggests that MG-2477 does not impair the viability of differentiated neuronal cells, but specifically targets undifferentiated cancer cells.

In parallel to flow cytometric analyses we also analyzed the viability of MG-2477-treated SH-EP and NB15 cells by an AlamarBlue assay. Viability was significantly more reduced than what we expected from flow cytometric analyses (Figure 1C). When analyzing the morphology of MG-2477-treated SH-EP cells by live cell fluorescence microscopy, we observed cockling and detachment of cells already after 3 hours, which continued up to 24 hours when hardly any attached, viable cells were left (Figure 1E). Nuclei fragmentation, visualized by Hoechst33342 staining, was rarely seen, which was consistent with relative low numbers of fragmented nuclei observed by propidium iodide (PI) flow cytometry. From the combined data we conclude that MG-2477 rapidly induces morphological changes and cell detachment in neuroblastoma cells, which eventually leads to cell death.

### MG-2477 induces formation of autophagosomes

**Figure 2:**
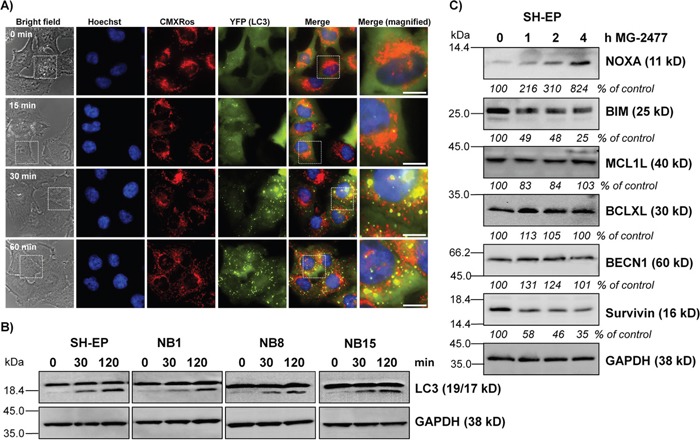
MG-2477 induces rapid and extensive autophagosome formation **(A)** SH-EP/YFP-LC3 cells were treated with 50 nM MG-2477. Autophagosome formation was monitored via live-cell microscopy up to one hour. Mitochondria were stained with MitoTrackerRed/CMXRos (300 nM), nuclei were stained with Hoechst33342 (100 ng/ml). Bar is 10 μm. **(B)** SH-EP, NB1, NB8, and NB15 cells were incubated for 30 and 120 minutes with 50 nM MG-2477. Cell lysates were subjected to immunoblot analyses for LC3 conversion. GAPDH served as loading control. **(C)** Immunoblot analyses of NOXA, BIM, MCL1L, BCLXL, BECN1, and Survivin expression after treatment of SH-EP cells for the times as indicated with 50 nM MG-2477. GAPDH served as loading control. Intensities of protein bands were quantified by densitometry, untreated cells were set as 100%.

As anti-mitotic drugs, including MG-2477 [10, 11, 34] have been reported to cause cell death associated with autophagy we investigated in a next step also the formation of autophagosomes during MG-2477 treatment and infected SH-EP, NB1, NB8, and NB15 cells with a retroviral vector coding for EYFP-LC3. Live cell fluorescence imaging of SH-EP/YFP-LC3 (Figure 2A), NB1/YFP-LC3, NB8/YFP-LC3, and NB15/YFP-LC3 cells (Supplementary Figure 3) demonstrates a rapid and extensive formation of autophagosomes already 30 minutes after MG-2477 addition in SH-EP cells and after one hour in NB1, NB8, and NB15 cells. Autophagosome formation was accompanied by the conversion of MAP1ALC3/LC3-I to LC3-II as analyzed by immunoblot (Figure 2B). To assess autophagic flux we overexpressed a DsRed-LC3-GFP fusion protein in SH-EP cells to visualize both, autophagosome formation and further fusion of autophagosomes with lysosomes in the presence or absence of BafilomycinA1 (BafA), an inhibitor of autophagosome-lysosome fusion [35]. As shown in Supplementary Figure 4, MG-2477 promotes autophagolysosome formation, which can be readily blocked by BafA. This implies that the large number of autophagosomes observed after 30 minutes of treatment results from increased *de novo* autophagosome formation and is not the consequence of autophagosome accumulation due to reduced fusion between autophagosomes and lysosomes. One essential trigger and key player of autophagosome formation is BECN1 which is normally bound to and thereby inactivated by members of the BCL2 protein family and by the inhibitor of apoptosis protein Survivin in healthy cells [16, 36–38]. We therefore analyzed the steady state expression of different pro- and anti-apoptotic proteins during MG-2477 treatment. Immunoblot analyses revealed that MG-2477 leads to a rapid decrease of Survivin, starting already after one hour. At the same time the pro-apoptotic BH3-only protein NOXA increases continuously, whereas BIM that sequesters BECN1 at dynein light chains [16] was repressed (Figure 2C and Supplementary Figure 5). MCL1, BCLXL and BECN1 levels remained largely unaffected during MG-2477 treatment. Interestingly, NOXA was recently described as rate-limiting BH3-only protein in the regulation of mitotic cell death [39] and Survivin was found to be degraded during autophagy in neuroblastoma [38]. Together, these results suggest that MG-2477 induces an immediate early autophagic response associated with increased expression of the BH3-only protein NOXA, repression of BIM and anti-apoptotic Survivin.

### NOXA displaces BECN1 from BCLXL and contributes to MG-2477-induced cell death

**Figure 3:**
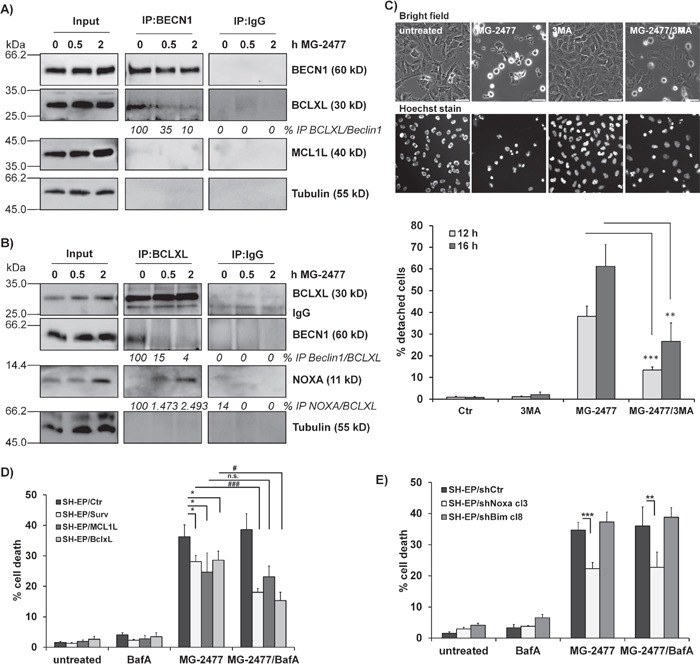
MG-2477-induced cell death depends on re-organization of pro- and anti-apoptotic proteins Co-immunoprecipitation of 1 μg BECN1 **(A)**, BCLXL **(B)**, or IgG control from SH-EP cells after treatment with 50 nM MG-2477 for 30 minutes and two hours. Input-lysates and precipitates were subjected to immunoblot analyses with antibodies directed against BCLXL, NOXA, MCL1 and BECN1. Tubulin served as loading control. **(C)** Live-cell microscopy of SH-EP cells treated with 50 nM MG-2477 for 12 and 16 hours alone or in combination with 0.5 mM 3MA (preincubated for 30 minutes). Nuclei were stained with Hoechst33324 (100 ng/ml). Pictures show representative images after 16 hours treatment. For quantification, 120 to 500 cells per treatment were counted and analyzed for detachment and nuclear fragmentation. Shown is the mean of four independent experiments. Statistical differences were assessed by unpaired t-test between MG-2477 and 3MA+MG-2477 treatment (***P<0.001; **P<0.01). SH-EP/Ctr, SH-EP/Surv, SH-EP/MCL1L and SH-EP/BclxL **(D)** or SH-EP/shCtr, SH-EP/shNoxa cl3 and SH-EP/shBim cl8 **(E)** cells were treated with 50 nM MG-2477 alone or in combination with 100 nM BafA (preincubated for 30 minutes) for 24 hours and subjected to PI-FACS analyses. Shown is the mean of four independent experiments. Statistical differences were assessed by unpaired t-test (significantly different between control and genetically modified cells ***P<0.0001; **P<0.01; *P<0.05; significantly different between MG-2477 and MG2477+BafA treated cells ###P<0.0001; #P<0.05).

**Figure 4:**
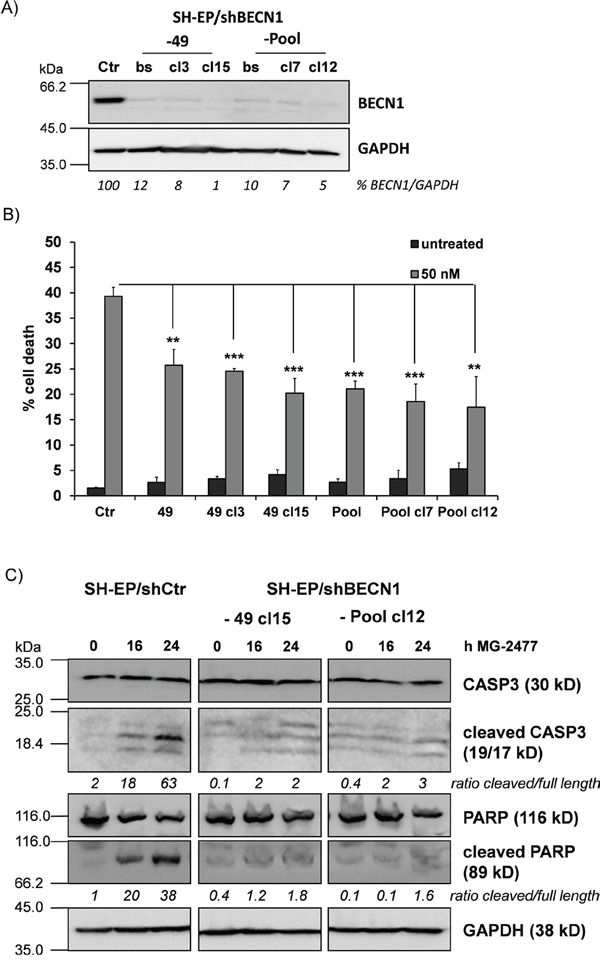
BECN1 is critical for MG-2477-induced cell death **(A)** Knockdown of BECN1 was verified by immunoblot in SH-EP cells infected with either a single short-hairpin sequence (-49) or a mixture of three individual short-hairpin sequences (-Pool). From the developed bulk-selected (bs) cell lines further individual clones (49: cl3, cl15; Pool: cl7, cl12) were raised. GAPDH served as loading control. **(B)** SH-EP/shCtr, SH-EP/shBECN1-49 cl3 and cl15 as well as SH-EP/shBECN1-Pool and -Pool cl7 and -Pool cl12 cells were treated for 24 hours with MG-2477 (50 nM). Cell death was assessed by PI staining of nuclei *via* flow cytometry. Shown is the mean of four independent experiments. **P<0.01; ***P<0.001. **(C)** SH-EP/shCtr, SH-EP/shBECN1-49 cl15 and SH-EP/shBECN1-Pool cl12 cells were treated with 50 nM MG-2477 for 16 and 24 hours. Cell lysates were subjected to immunoblot analyses for CASP3 and PARP cleavage. GAPDH served as loading control. Densitometry was done using Labworks software and expressed as cleaved/full length ratio.

**Figure 6:**
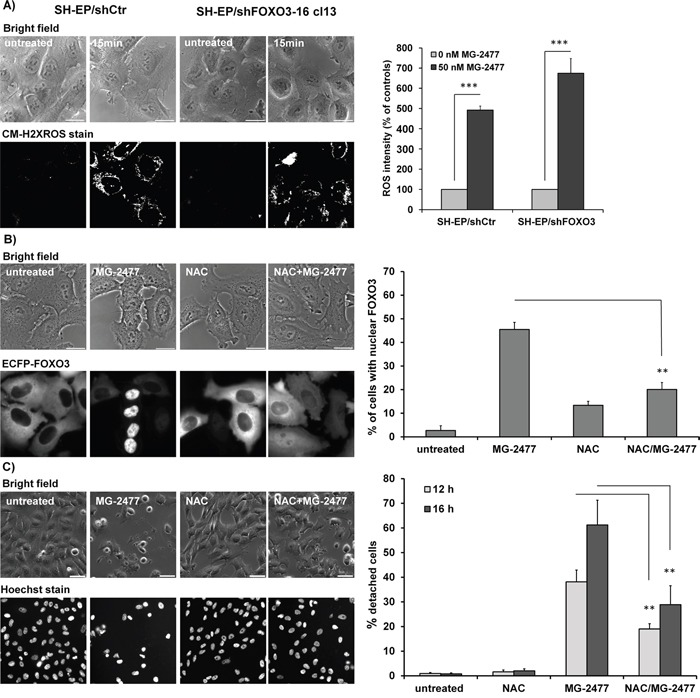
MG-2477-induced ROS accumulation occurs downstream of PI3K class III activation and is independent of FOXO3 **(A)** ROS were measured by CM-H2XROS staining (500 nM) of SH-EP/shCtr and SH-EP/shFOXO3-16 cl13 cells after treatment with 50 nM MG-2477 for 15 minutes. For the quantification of cellular ROS intensity more than five pictures (more than 30 cells) were densitometrically quantified (***P<0.001). **(B)** Live-cell analyses of SH-EP/ECFP-FOXO3 cells after treatment with 50 nM MG-2477 for 60 minutes alone or in combination with 5 mM NAC (preincubated for 30 minutes). Shown is the mean of three independent experiments, statistical differences were assessed by unpaired t-test (**P<0.01). Bar is 20 μm. **(C)** Live-cell microscopy of SH-EP cells treated with 50 nM MG-2477 for 12 and 16 hours alone or in combination with 5 mM NAC (preincubated for 30 minutes). Nuclei were stained with Hoechst33324 (100 ng/ml). Pictures show representative images after 16 hours treatment. Bar is 50 μm. For quantification between 140 and 500 cells *per* treated glass slide were counted and analyzed for detachment and fragmented nuclei. Shown is the mean of four independent experiments. Statistical differences were assessed by unpaired t-test between MG-2477 and NAC+MG-2477 treatment, (**P<0.01).

In a next step we determined whether autophagy induction by MG-2477 is critically influenced by NOXA as NOXA may neutralize the autophagy-inhibiting capacity of pro-survival BCL2-proteins. The pro-survival BCL2 proteins BCLXL as well as MCL1 which are both bound by NOXA in neuroblastoma cells [40] inhibit autophagy by sequestration of BECN1 [41]. Therefore we precipitated endogenous BECN1 from MG-2477-treated SH-EP cells and analyzed BECN1-associated candidate proteins in neuroblastoma cells. As shown in Figure 3A, in untreated cells BCLXL binds to BECN1 and this interaction is markedly reduced already within 30 minutes in the presence of MG-2477. In contrast, no interaction between BECN1 and MCL1 was detected in SH-EP cells. *Vice versa* immunoprecipitation of BCLXL confirmed that 30 minutes after MG-2477-addition BECN1 disappears from BCLXL protein complexes, whereas the amount of bound NOXA strongly increases. This supports the hypothesis that early during MG-2477-treatment BECN1 is displaced from BCLXL by increased amounts of cellular NOXA, which triggers autophagy initiation in neuroblastoma cells (Figure 3B). To determine whether this induction of autophagy is necessary for the further cytotoxic effects of MG-2477, we monitored cell morphology/detachment as well as Hoechst33342-stained nuclei by live cell microscopy in the presence or absence of the autophagy inhibitor 3-Methyladenine (3MA) which inhibits class III PI3-kinases and thereby blocks the first steps of the autophagic process. As shown in Figure 3C and Supplementary Figure 6 3MA effectively prevents the formation of autophagosomes and rescues SH-EP cells from detachment/dying after treatment with 50 nM MG-2477. Together, these results suggest the sequestration of anti-apoptotic BCL2 proteins by NOXA and the release of BECN1 as an inducer of autophagy and potential cell death trigger. Therefore, in a next step we knocked down BECN1 in SH-EP and NB15 cells [38] by stable expression of shRNAs directed against BECN1 (Figure 4 and Supplementary Figure 7). As shown in Figure 4A, the almost complete repression of BECN1 strongly reduced cell death in SH-EP (Figure 4B) and NB15 cells (Supplementary Figure 7A) as measured by flow cytometry and also reduced CASP3 as well as PARP cleavage up to 24 hours post addition of MG-2477 (Figure 4C). Also ROS accumulation, as demonstrated in Figure 6A, was completely inhibited in SH-EP/shBECN1-49 cl15 and SH-EP/shBECN1-Pool cl12 cells (Supplementary Figure 7B). This implies that BECN1 is one critical player in the death-inducing signaling cascade triggered by MG-2477. To further analyze the relevance of the BCL2-protein/BECN1 and Survivin/BECN1 complexes in the initial phase of MG-2477-induced cell death, we overexpressed Survivin (SH-EP/Surv), MCL1L (SH-EP/MCL1L) and BCLXL (SH-EP/BclxL) and analyzed the effect of these proteins on MG-2477-induced cell death. Since both, Survivin and BCLXL, were described as targets for autophagolysosomal degradation [38, 42], we additionally pretreated the cells with BafA. As demonstrated in Figure 3D the ectopic expression of all three pro-survival proteins lowered MG-2477-induced apoptosis. Consistent with their degradation by autophagy, co-treatment with BafA further enhanced the inhibitory effects of Survivin and BCLXL, but not of MCL1. To directly assess whether NOXA is the critical trigger, its induction was prevented by shRNA expression: Knockdown of NOXA (Supplementary Figure 8A) markedly reduced MG-2477-induced cell death (Figure 3E), CASP3 and PARP cleavage (Supplementary Figure 8B), whereas knockdown of BIM [43] had no effect on cell death induction. This implies that although BIM might sequester BECN1 at dynein light chains [16], its repression does not contribute to autophagy and subsequent death induction. Of note, the knockdown of NOXA lowered MG-2477-induced cell death to a similar extent as ectopically expressed MCL1, without being affected by the co-treatment with BafA (Figure 3E). The combined data suggest that the induction of NOXA by MG-2477 (Figure 2C) displaces BECN1 from anti-apoptotic BCLXL, which increases the amount of unbound BECN1 (Figure 3A and 3B) and thereby triggers autophagy. Both, NOXA and BECN1 are essential players in this initial phase and their knockdown strongly reduces the efficacy of MG-2477. Transgenic expression of MCL1, however, indirectly slows down BECN1 activation by sequestering NOXA away from BCLXL, as NOXA binds MCL1 with higher affinity than BCLXL in neuroblastoma cells [44].

### MG-2477 inhibits the PI3K-PKB axis and causes FOXO3 activation

**Figure 5:**
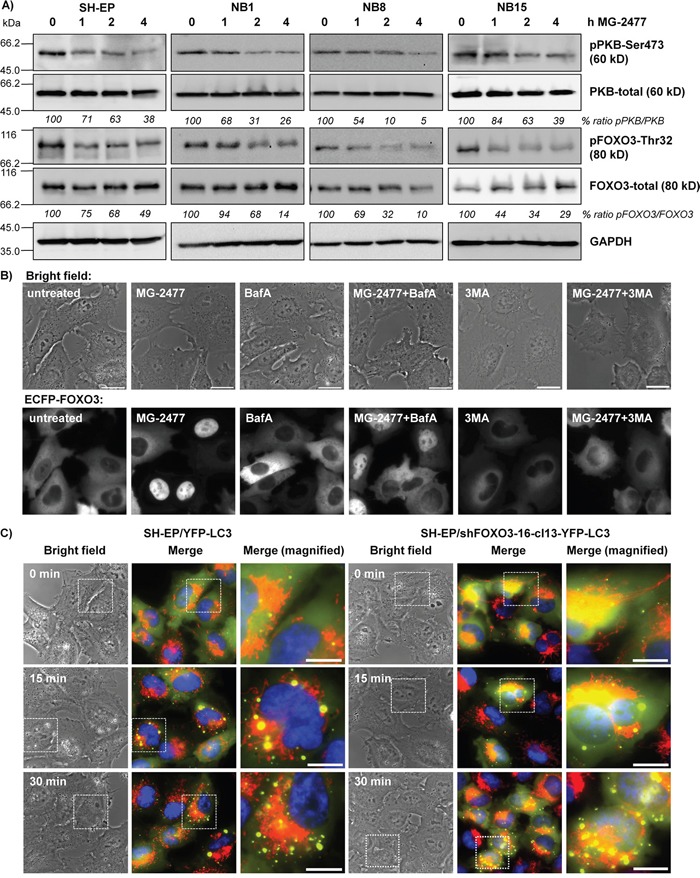
MG-2477-treatment causes PKB de-phosphorylation and nuclear accumulation of FOXO3 **(A)** SH-EP, NB1, NB8, and NB15 cells were treated for up to four hours with 50 nM MG-2477. Cell lysates were subjected to immunoblot analyses with antibodies directed against PKB (Ser473 and total) and FOXO3 (Thr32 and total). GAPDH served as loading control. **(B)** Representative live-cell analyses of SH-EP/ECFP-FOXO3 cells after treatment with 50 nM MG-2477 for up to 60 minutes alone or in combination with 0.5 mM 3MA or 100 nM BafA (preincubated for 30 minutes). **(C)** SH-EP/YFP-LC3 and SH-EP/shFOXO3-16-cl13-YFP-LC3 cells were treated with 50 nM MG-2477. Autophagosome formation was monitored *via* live-cell microscopy up to 30 minutes. Mitochondria were stained MitoTracker Red/CMXRos (300 nM), nuclei were stained with Hoechst33342 (100 ng/ml). Bar is 10 μm.

Autophagy is regulated via class II and class III PI3-kinases [45]. One critical downstream target of the PI3K is the PKB and this survival pathway is frequently hyperactivated in neuroblastoma [4], which among other targets inactivates the transcription factor FOXO3 [46]. Therefore we analyzed the effect of MG-2477 on the PKB - FOXO3 signaling axis. Within one hour of MG-2477 treatment, the phosphorylation of PKB at serine473 (Ser473) was decreased to 71% and after four hours to 38% in SH-EP cells and almost identical regulations were also observed in NB1, NB8, and NB15 cells upon MG-2477 treatment (Figure 5A). Simultaneously, also the phosphorylation of FOXO3 was significantly reduced at threonine32 (Thr32) to 49% in SH-EP cells and down to even 10% in NB8 cells, suggesting FOXO3 activation under these conditions. We also analyzed the phosphorylation of c-jun-N-terminal kinase (MAPK8/JNK) which was also shown to participate in autophagy induction after stress signaling [47]. JNK as well as phospho-JNK-threonine183/tyrosine185 (Thr183/Tyr185) were slightly induced after MG-2477 treatment, but we did not observe a significant change in the phospho-JNK/JNK ratio (Supplementary Figure 9), therefore we excluded JNK as critical regulator of MG-2477-induced death.

To study, whether reduced PKB activity and reduced FOXO3-phosphorylation during MG-2477 treatment also leads to FOXO3 nuclear accumulation we determined the subcellular localization of an ECFP-tagged FOXO3 allele by live cell fluorescence microscopy. In line with dephosphorylation of FOXO3 at Thr32 after one hour of MG-2477 treatment, we observed nuclear ECFP-FOXO3 accumulation after 60 minutes (Figure 5B). Since FOXO3 also regulates the transcription of genes which are involved in autophagy-regulation, like LC3 and BNIP3 [48, 49] and to clarify the sequence of events which activate FOXO3 after MG-2477 treatment, we analyzed ECFP-FOXO3 translocation also after pre-incubation with either 3MA or BafA. Inhibition of the formation of autophagolysosomes by BafA had only a minor, not significant effect on the nuclear accumulation of FOXO3 (Figure 5B and Supplementary Figure 10). Upstream inhibition of autophagy at the level of PI3K class III/BECN1 by 3MA, however, significantly reduced the number of cells with nuclear FOXO3 after MG-2477 treatment (Figure 5B and Supplementary Figure 10). As a control, we next generated YFP-LC3-expressing neuroblastoma cells with FOXO3 being knocked down by shRNA technology (SH-EP/shFOXO3-16-cl13-YFP-LC3 cells) to monitor autophagosome formation. Knockdown of FOXO3 did not affect the formation of autophagosomes during MG-2477 treatment (Figure 5C), supporting the results from live cell fluorescence imaging that activation of FOXO3 occurred downstream of PI3K class III/BECN1. Together, our results suggest that FOXO3 activation does not trigger immediate early autophagy, but by its later activation may contribute to the perpetuation of autophagy *via* induction of autophagy key regulators and to cell death.

### Early ROS accumulation in MG-2477-treated cells occurs downstream of PI3K class III activation and is independent of FOXO3

FOXO proteins are regulated by increased cellular reactive oxygen species (ROS) during stress signaling [50], but cause also ROS accumulation in neuroblastoma cells [43]. Thus we next assessed whether MG-2477 treatment is associated with increased cellular ROS and investigated how ROS relate to autophagy, FOXO3 activation and subsequent cell death induction. As shown in Figure 6A, MG-2477 treatment caused elevation of cellular ROS levels as early as 15 minutes *post* addition of the drug. This highly significant accumulation of ROS in the early phase of MG-2477 treatment was also visible in NB1, NB8, and NB15 cells (Supplementary Figure 11). The knockdown of endogenous FOXO3 in SH-EP/shFOXO3-16-cl13 cells (Supplementary Figure 15A) did not influence ROS production (Figure 6A), suggesting that neither autophagosome formation (as shown in Figure 5C) nor ROS accumulation in this early phase of MG-2477 treatment depend on FOXO3. However, pre-treatment of SH-EP/ECFP-FOXO3 cells with the ROS inhibitor N-acetyl-L-cysteine (NAC) for 30 minutes reduced nuclear accumulation of FOXO3 in response to MG-2477 (Figure 6B) and NAC treatment also rescued MG-2477-treated SH-EP cells from detaching and dying (Figure 6C), comparable to 3MA (Figure 3C). To investigate this effect in more detail we studied the impact of NAC on autophagosome formation and on the regulation of NOXA, BIM, Survivin, and BECN1 in MG-2477 treated/untreated cells. Rather surprisingly, NAC efficiently prevented the formation of autophagosomes (Supplementary Figure 12), inhibited CASP3 cleavage (Supplementary Figure 13B) and attenuated, but did not prevent the regulation of NOXA and Survivin during MG-2477 treatment (Supplementary Figure 13A). This suggests that ROS are generated in the initial phase of cell death and contribute to the perpetuation of the whole process. As shown in Supplementary Figure 7B, the knockdown of BECN1 completely prevented ROS accumulation. The combined data from these experiments suggest that ROS accumulation is downstream of BECN1 and that FOXO3 is activated downstream of cellular ROS accumulation and does not affect autophagosome formation in this initial phase of MG-2477-induced death.

**Figure 7:**
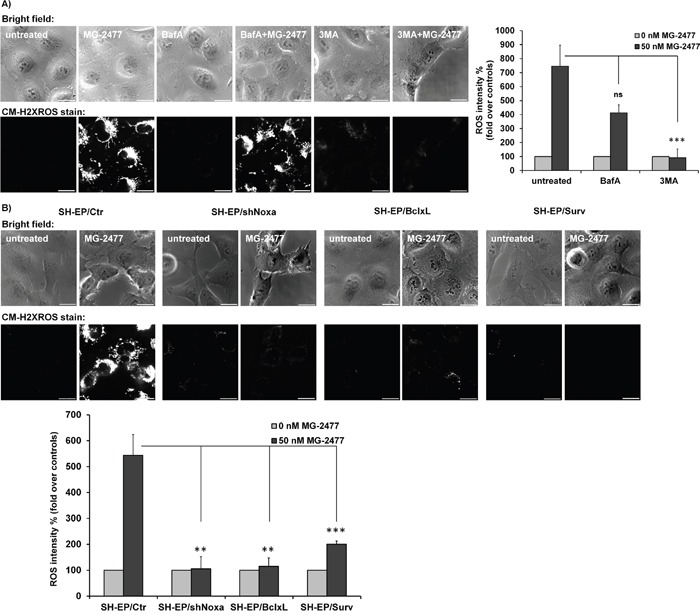
ROS accumulation is triggered downstream of BCL2 rheostat/BECN1 **(A)** Cellular ROS levels were assessed by CM-H2XROS staining (500 nM) after treatment of SH-EP cells with 50 nM MG-2477 alone or in combination with 100 nM BafA, or 0.5 mM 3MA for 30 minutes. Bar is 20 μm. **(B)** SH-EP/Ctr, SH-EP/shNoxa cl3, SH-EP/BclxL, and SH-EP/Surv cells were treated with 50 nM MG- 2477 for 30 minutes and analyzed for ROS by CM-H2XROS staining (500 nM). For the quantification of cellular ROS intensity, the cells of three independent experiments (in each experiment three to four micrographs, more than 30 cells per experiment) were densitometrically analyzed using Axiovert Software (Zeiss, Vienna) and statistical differences were assessed by unpaired t-test (***P<0.001; **P<0.01).

To further analyze how ROS accumulation is connected to autophagy initiation, the cells were pre-incubated for 30 minutes with either 3MA or BafA before MG-2477 was added for additional 30 minutes (Figure 7A). Whereas BafA did not show a statistically significant protective effect, 3MA almost completely prevented ROS accumulation suggesting that ROS accumulation can be prevented by inhibition of the PI3K class III/BECN1 complex. This is in line with the pronounced effect of BECN1 knockdown demonstrated in Supplementary Figure 7B and defines the time point of ROS formation downstream of the PI3K-III and BECN1 activation, but upstream of autophagosome formation. Since BCLXL and MCL1 are both, directly or indirectly, involved in BECN1 inactivation, we also assessed the influence of the ectopic expression of these pro-survival BCL2 proteins on ROS formation. In line with competitive displacement of BECN1 from BCLXL by NOXA, the ectopic expression of BCLXL as well as MCL1 and the knockdown of NOXA expression blocked ROS formation (Figure 7B and Supplementary Figure 14). Also Survivin overexpression, which reduced MG-2477-induced apoptosis in an autophagy-dependent manner, efficiently reduced ROS formation (Figure 7B), either by shutting down mitochondrial respiration at complex I or by sequestering BECN1, as we have described before [5, 38, 51].

### FOXO3-activation by MG-2477 enhances cell death signaling

**Figure 8:**
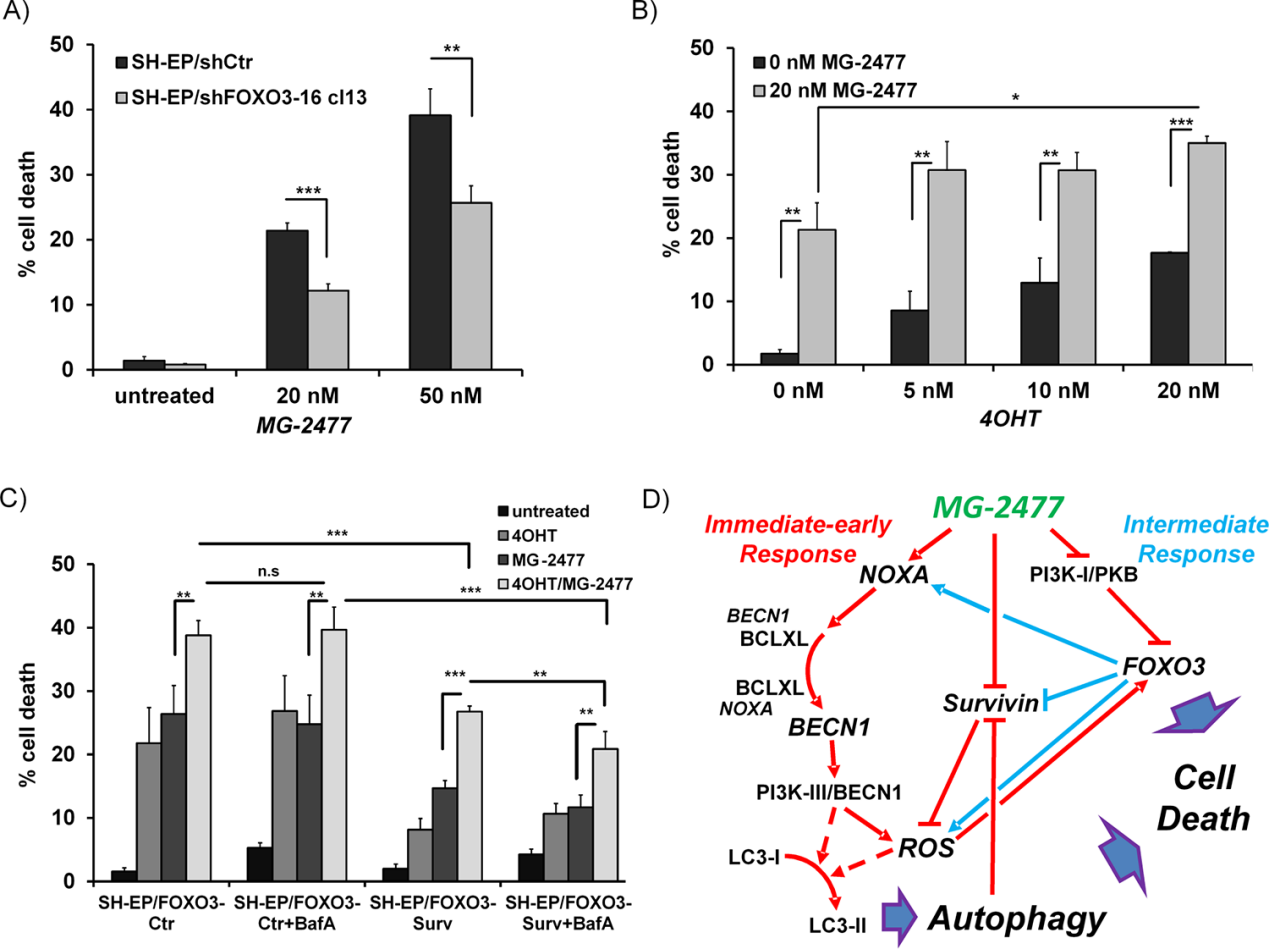
FOXO3-activation enhances MG-2477-induced cell death **(A)** SH-EP/shCtr and SH-EP/shFOXO3-16 cl13 cells were incubated with 20 or 50 nM MG-2477 for 24 hours and cell death was measured by PI-FACS analyses (shown is the mean of three independent experiments). **(B)** SH-EP/FOXO3 cells were treated with 20 nM MG-2477 alone or in combination with increasing amounts of 4OHT (5 to 20 nM) for 24 hours and analyzed for PI-stained nuclei by flow cytometry. **(C)** SH-EP/FOXO3-Ctr and SH-EP/FOXO3- Surv cells were pre-incubated with 20 nM 4OHT (24 hours) and/or 100 nM BafA (30 minutes) before addition of 35 nM MG-2477 for another 24 hours. Shown is the mean of four independent experiments. Statistical difference in all experiments was assessed by unpaired t-test (***P<0.001; **P<0.01, *P<0.05). **(D)** Schematic model of the molecular events triggered by MG-2477 treatment in neuroblastoma cells: In the immediate early phase (red lines), MG-2477 treatment increases NOXA expression, which replaces BECN1 from BCLXL. This triggers ROS downstream of the PI3K-III/BECN1 complex, which results in LC3 conversion, autophagosome formation and autophagic flux. In parallel, Survivin steady state levels decrease, which according to earlier studies from our lab further promotes ROS accumulation. MG-2477 represses the PI3K-I/PKB pathway leading to FOXO3 nuclear accumulation (intermediate phase, blue lines) and to further activation of cellular ROS. In this cell system we have demonstrated before that FOXO3 among other death-related targets induces NOXA and represses Survivin and thereby further perpetuates authophagy and drives the cells into cell death.

Besides the reassortment of BECN1, our results suggest a critical role for NOXA, BCLXL, and Survivin in MG-2477-induced cell death. All three proteins are direct or indirect targets of the transcription factor FOXO3 in neuroblastoma cells [43, 46, 52, 53] implicating that, if not in the initiation phase, the secondary induction and repression of these key players by FOXO3 might contribute to cell death. Since MG-2477 triggers nuclear accumulation of FOXO3 (Figure 5B and 6B), we next tested the relevance of FOXO3 for MG-2477-induced cell death. SH-EP/shFOXO3-16 cl13 cells were subjected to MG-2477 treatment and analyzed for cell death. Knockdown of FOXO3 in SH-EP cells reduces cell death from 21.4% to 12.2% after 24 hours and from 40.3% to 23.7% after 48 hours, suggesting that FOXO3 significantly participates in cell death induction (Figure 8A). On the level of NOXA, BIM, and Survivin steady state expression it becomes evident that reduction of FOXO3 significantly attenuates gene regulation of NOXA and Survivin, whereas the downregulation of BIM or steady state expression of BECN1 was not affected by FOXO3 knockdown (Supplementary Figure 15B). The putative role of FOXO3 as an “apoptosis amplifier” during MG-2477 treatment is shown in immunoblots measuring CASP3 and PARP cleavage (Supplementary Figure 15C): at 16 hours post addition of MG-2477 hardly any difference is visible between SH-EP/shCtr and SH-EP/shFOXO3-16 cl13 cells, whereas at 24 hours the cleavage of CASP3 and of PARP is significantly reduced in the FOXO3 knockdown cells.

In a next step, as *vice versa* experiment, we also tested whether the activation of ectopic FOXO3 affects MG-2477-induced cell death. For that purpose we used SH-EP/FOXO3 cells that express an ectopic, phosphorylation-independent FOXO3(A3)ERtm fusion protein [46] and treated these cells with increasing amounts of 4-hydroxy-tamoxifen (4OHT). FOXO3-activation by 4OHT enhances MG-2477-induced cell death from 21.3% to 30.7-35.0% (Figure 8B).

We recently identified the FOXO3 target gene Survivin as both, a critical regulator and a target of autophagosomal degradation [38, 51]. Survivin was rapidly downregulated during MG-2477 treatment and this repression is attenuated in FOXO3-knockdown cells (Supplementary Figure 15B). However, when overexpressed, Survivin counteracts MG-2477-induced cell death raising the question, whether this protective effect can be overcome by FOXO3-mediated repression of Survivin and if increased cell death after FOXO3 activation depends on Survivin levels. Therefore we pre-treated either SH-EP/FOXO3-Ctr or SH-EP/FOXO3-Surv cells for 24 hours with 20 nM 4OHT to activate FOXO3 and then added MG-2477 (35 nM) alone or in combination with BafA. As demonstrated in Figure 8C Survivin-expressing cells are protected from FOXO3-induced cell death as well as show reduced apoptosis after MG-2477 treatment. Combined treatment of MG-2477 and 4OHT, however, induces cell death in SH-EP/FOXO3-Surv cells to the same amount as in SH-EP/FOXO3-Ctr cells (26.4% and 26.8%). Co-treatment with BafA slightly lowers this effect to 20.9%, suggesting that inhibition of autophagy-mediated Survivin degradation partially counteracts MG-2477-induced cell death. Immunoblot analyses of SH-EP/FOXO3-Surv cells reveal that the additional activation of the ectopic 4OHT-regulated FOXO3 allele causes a rapid decay of Survivin steady-state levels (Supplementary Figure 16), explaining the increase of cell death. The combined data support the model that ROS-driven FOXO3 activation enhances the cytotoxic effect of MG-2477 by modulating the regulation of NOXA, Survivin and possibly other death regulators, which triggers additional death pathways and eventually drives the tumor cell into cell death.

## DISCUSSION

Anti-mitotic drugs have been used for a long time in cancer therapy but, as also true for almost all other chemotherapeutics, their therapeutic efficacy is challenged by cancer cells that develop drug resistance. Therefore much effort is put into the improvement and patient-oriented re-design of such “old” drugs. In the present study we demonstrate that MG-2477, a derivate of phenylpyrroloquinolinone, efficiently kills neuroblastoma cells without affecting differentiated neuronal cells. The cytotoxic effects of MG-2477 have also been demonstrated in liver, ovarian or non-small lung carcinoma cell lines [11, 27]. However, anti-mitotic drugs are thought to be inactive in cancer cells with high levels of active PKB [30], a situation present in most malignant neuroblastoma cells [54, 55]. Since MG-2477 was shown to reduce PKB phosphorylation [11], we hypothesized that it might be a suitable drug for neuroblastoma therapy. To analyze the effect of MG-2477 on the survival of neuroblastoma cells, we chose a set of cell lines that are derived from different tumor stages [31, 40] and vary in PKB expression and PKB activity (phosphoPKB/PKB ratio from 1.2 to 8.6, Supplementary Figure 1B) and MYCN status [56, 57]. MG-2477 treatment resulted in similar cell death induction and reduction of viability in all tested neuroblastoma cell lines, independent of the stage derived from, the PKB or the MYCN status (Figure 1 and Supplementary Figure 1B) [31, 40, 46, 58]. As a control for the selectivity on cancer cells, we further tested MG-2477 on all-trans retinoic acid (RA) differentiated NB8 and NB15 cells and on NGF-differentiated PC12 cells. All three cell lines were highly resistant to higher concentrations of the drug than undifferentiated cells (Supplementary Figure 2), suggesting that this compound selectively acts on undifferentiated, malignant neuronal cells and shows low neurotoxicity on differentiated neurons.

The observed reduction of cell viability induced by MG-2477 (Figure 1C) was accompanied by detachment of neuroblastoma cells after 15 hours without typical DNA-fragmentation (Figure 1E), although at 24 and 48 hours DNA fragmentation, Annexin V positivity, CASP3 and PARP cleavage were detectable (Figure 1A, 1B and 1D). We therefore investigated the initial phase of MG-2477 treatment and uncovered that death induction was preceded by immediate-early autophagy, with autophagosome formation and LC3-conversion being already extensively detectable 15 to 30 minutes after MG-2477 treatment (Figure 2A and 2B, Supplementary Figures 3 and 4). In this immediate early phase we observed the induction of the BH3-only protein NOXA, repression of the BECN1-scavenging BH3-only protein BIM [16, 59] and repression of the BECN1-interacting Survivin (Figure 2C) [38, 51]. This differs from results of Viola and colleagues who observed autophagy simultaneously to signs of apoptosis in MG-2477-treated lung carcinoma cells at later time points [11]. Since increasing amounts of NOXA co-immunoprecipitated with BCLXL and BECN1 in parallel disappeared from BCLXL during treatment, these re-assortments suggest that autophagy is induced *via* increased levels of non-sequestered BECN1 that is displaced from BCLXL by NOXA (Figure 3A and 3B). Further flow cytometric analyses support this assumption of a critical role of NOXA in autophagy and subsequent cell death induction, since ectopic expression of the NOXA binding partners MCL1 and BCLXL, as well as NOXA and BECN1 knockdown reduced MG-2477-induced death (Figures 3D and 3E, Figure 4, Supplementary Figures 7A and 8B). In our model NOXA therefore induces autophagy by binding to BCLXL, which displaces BECN1 from BCLXL. Autophagy triggered by this re-assortment further causes degradation of Survivin, a recently identified key-player of autophagy in neuroblastoma [38], which further releases BECN1 from Survivin/BECN1 [38] complexes and leads to a feed-forward enhancement of autophagic signaling. This explains why ectopic expression of BCLXL or Survivin efficiently counteract autophagy induction similar to ectopically expressed MCL1, which sequesters NOXA with higher affinity than BCLXL [44] and also prevented ROS accumulation in response to MG-2477 treatment (Supplementary Figure 14). 3MA prevented autophagy (Supplementary Figure 6) and rescued SH-EP cells from MG-2477-induced death (Figure 3C), which raises the question how autophagy contributes to cell death induction. A crosstalk between autophagy and cell death is in line with a previous report on another mitotic inhibitor, where parallel induction of autophagy and apoptosis after treatment with paclitaxel was observed [34]. Neither autophagy nor apoptosis inhibitors alone were able to rescue cells from paclitaxel-induced death suggesting that one pathway fills in for the other in case of failure to eliminate cells. In another recent paper on the ALK inhibitor entrectinib, Aveic et al demonstrated that autophagy reduces the efficacy of ALK inhibition and that partial resistance to this ALK inhibitor can be overcome by autophagy-inhibition *via* chloroquine [60]. However, in neuroblastoma cells, autophagy by MG-2477 is induced rapidly after treatment (within 30 minutes, Figure 2), while cell death occurred delayed between 24 and 48 hours. As BECN1 knockdown and 3MA efficiently reduced cell death, in neuroblastoma cells a sequential process of autophagy followed by death induction can be proposed. This also fits to the observation that FOXO3 activation is downstream of initial autophagy signaling, since 3MA was able to prevent FOXO3 translocation (Figure 5B) and ROS accumulation after MG-2477 treatment (Figure 7A), whereas the late autophagy inhibitor BafA failed to do so (Figure 5B and 7A). Knockdown of endogenous FOXO3 had no effect on autophagosome formation by MG-2477 (Figure 5C) further supporting this notion, but attenuated NOXA and Survivin regulation (Supplementary Figure 15B) and reduced CASP3 and PARP cleavage at 24 hours post addition of the drug (Supplementary Figure 15C) as well as DNA fragmentation (Figure 8A). This suggests that FOXO3 significantly contributes to the apoptotic part of MG-2477-induced cell death. One critical trigger for FOXO3 activation besides reduced PKB survival signaling is the accumulation of cellular ROS, which are frequent byproducts of autophagy. Some substances such as compound K, safingol or buralin were described to induce autophagy in a ROS-dependent manner [61–63]. Also in the case of MG-2477 the ROS inhibitor NAC reduced autophagosome formation (Supplementary Figure 12). Interestingly, when we analyzed ROS levels in BECN1-knockdown cells (Supplementary Figure 7B) or after pre-incubation with 3MA and BafA, knockdown of BECN1 and 3MA treatment efficiently prevented ROS accumulation (Figure 7A). This suggests that ROS accumulation is triggered downstream of the PI3K-III/BECN1 complex and might be further amplified as a consequence of autophagy-dependent degradation of Survivin [38] as well as by FOXO3 activation [43]. Therefore, ROS are rather a byproduct of autophagy initiation and not the initial cause, but likely further perpetuate this initial autophagy, a notion that is further supported by the attenuation of NOXA and Survivin regulation in NAC-treated cells (Supplementary Figure 13). In the case of MG-2477, the re-organization of the PI3K-III/BECN1 complex by induction of NOXA and decay of Survivin seems to be critical for the observed ROS accumulation that further shifts the signaling towards cell death induction, since it contributes to the activation of the transcription factor FOXO3 (Figure 5A, 5B and Figure 6B), which further enhances cell death, whereas knockdown of FOXO3 reduces death (Figure 8A, Supplementary Figure 15C). In this intermediate phase (Figure 8D) especially the FOXO3 target gene Survivin may play a critical role in pushing autophagic signaling toward cell death, as it was rapidly degraded after MG-2477 treatment and, when overexpressed, inhibited MG-2477-induced cell death in an autophagy-dependent manner (Figure 2C, Figure 8C, Supplementary Figure 16). This is consistent with our recent discovery that Survivin is a target for autophagosomal removal in neuroblastoma [38, 51] and our results are in line with data on another IAP, BIRC6/BRUCE, which is also degraded *via* autophagosomal removal [25]. Importantly, FOXO3-mediated repression of Survivin at least partially abrogated the death-protective effect of overexpressed Survivin (Figure 8C, Supplementary Figure 16). Since Survivin expression is frequently elevated in human high-stage neuroblastoma due to a gain of chromosome 17q [3], MG-2477 represents an interesting substance for the treatment of this disease, as it might allow to neutralize death-protection by elevated endogenous Survivin.

The combined data suggest autophagy induction and ROS accumulation as critical early events during MG-2477 treatment, whereas downstream of these initial events FOXO3 is activated, which is critical for cell death induction (summarized in Figure 8D). By uncovering the sequence of regulations which finally lead to the elimination of cancer cells we were able to identify BECN1, NOXA and Survivin as initial key players in MG-2477-induced cell death. Especially the repressive effect of MG-2477 on Survivin will be important in novel treatment strategies to overcome Survivin-mediated therapy-resistance in aggressive neuroblastoma.

## MATERIALS AND METHODS

### Cell lines, culture conditions and reagents

The human neuroblastoma cell lines SH-EP (kindly provided by N. Gross, Lausanne, Switzerland), IMR32, STA-NB1, STA-NB3, STA-NB4, STA-NB8, and STA-NB15 [31, 40] (gift of P. Ambros, St. Anna Children's Hospital, Vienna, Austria – these cells are named throughout the manuscript without STA), the acute lymphatic leukemia cell line CEM-C7H2-2c8, Phoenix™ packaging cells (kindly provided by G. Nolan, Standford) and all sublines were cultured in RPMI1640 (BioWhittaker, Belgium) containing 10% fetal calf serum (FCS, Gibco BRL, Paisley, GB), 100 U/ml penicillin, 100 μg/ml streptomycin and 2 mM L-glutamine (Lonza, Basel, Switzerland) at 5% CO_2_ and 37 °C in saturated humidity. HEK293T packaging cells for the production of lentiviral particles were cultured in DMEM (Invitrogen, Carlsbad, CA, USA). The pheochromocytoma cell line PC12 was cultivated in 10% horse serum (Gibco BRL, Paisley, GB) and 5% calf serum. For differentiation into neurons, PC12 cells were plated onto collagen-coated 24 well plates and after adhesion the medium was replaced by RPMI1640 containing 1x N2 Supplement (Life Technologies, Carlsbad, CA., USA). All cultures were routinely tested for mycoplasma contamination (Venor^R^GeM-mycoplasma detection kit, Minerva). All reagents were purchased from Sigma-Aldrich (Vienna, Austria) unless indicated otherwise. MG-2477 was produced as described in [27]. BafilomycinA1 was purchased from Enzo-Life-Science (Vienna, Austria).

### Production of retro- and lentiviruses for infection of neuroblastoma cells

Retro- and lentiviruses were produced as previously described [38, 43]. Briefly, viral vectors were transfected into amphotropic Phoenix packaging cells for retroviruses and into HEK293T cells (together with the packaging plasmid pCMV 8.74) for lentiviruses using lipofectamine2000 (Invitrogen, Carlsbad CA, USA). Supernatants were harvested two days after transfection and used to infect target cells. The following vectors have been described previously: pLIB-FOXO3(A3)ERtm-iresNeo, pLIB-BclxL-iresPuro, pLIB-MCL1L-iresPuro, pQ-tetH1-shBim-SV40-Puro, pQ-tetH1-shNoxa-SV40Puro, pLIB-ECFP-FOXO3wt-iresPuro, pLIB-Survivin-iresYFP, pLIB-MCS2-iresPuro, pLIB-MCS2-iresNeo, pQ-tetH1-SV40Puro and pQCXI-DsRed-LC3-GFP-puro [5, 35, 38, 40, 43, 46, 52, 64]. The lentiviral vectors coding for FOXO3-specific shRNA (pLKO1-shFOXO3-91616), for BECN1-specific shRNA (pLKO1-shBECN1-49, pLKO1-shBECN1-50, pLKO1-shBECN1-51) and the control vector pLKO.1 were obtained from Thermo Scientific (Huntsville, AL, USA) and Dharmacon (Lafayette, CO, USA), respectively. For expression of YFP-tagged LC3, mus-LC3 was amplified from pEYFP-C1-mus-LC3 (kindly donated from Prof. Noboru Mizushima, Tokyo Medical and Dental University). To generate the vector pLIB-EYFP-LC3-iresPuro the PCR-product was inserted into the EcoR1 and BamH1 site of the previously described pLIB-EYFP-MCL1_JAM_-iresPuro [44] thereby replacing MCL1_JAM_ by LC3.

### Genetically modified neuroblastoma cell lines

The cell lines SH-EP/Ctr, SH-EP/shCtr, SH-EP/Surv, SH-EP/FOXO3-Ctr, SH-EP/FOXO3-Surv, SH-EP/MCL1L, SH-EP/BclxL, SH-EP/shNoxa cl3, SH-EP/shBim cl8, and SH-EP/ECFP-FOXO3 have been described before [5, 40, 43, 46, 52]. The cell line SH-EP/shFOXO3-16-cl13 was generated by lentiviral infection with pLKO-shFOXO3-91616 supernatants. From the bulk selected cell line single clones were raised. The cell lines SH-EP/shBECN1-49 and -Pool were generated by lentiviral infection with pLKO-shBECN1-49 or a pool of three vectors (pLKO-shBECN1-49, -50, 51), respectively. From bulk-selected, infected cells single clones (SH-EP/shBECN1-49 cl3, cl15 or SH-EP/shBECN1-Pool cl7, cl12) were isolated. NB15/shBECN1-49 and NB15/shBECN1-51 cells were described before [38]. Retroviral supernatants of pLIB-EYFP-LC3-iresPuro were used to generate SH-EP/YFP-LC3 and SH-EP/shFOXO3-16-cl13-YFP-LC3 as well as NB1/YFP-LC3, NB8/YFP-LC3 and NB15/YFP-LC3 cells. The cell line SH-EP/dsRed-LC3-GFP was generated by retrovial infection of SH-EP cells with the plasmid pQCXI-DsRed-LC3-GFP-puro [35].

### Cell viability

Apoptosis was assessed by staining the cells with Annexin-V-FITC or propidium-iodide (PI) Triton-X-100 buffer using a CytomicsFC-500 Beckman Coulter as previously described [44]: 2×10^5^ cells were centrifuged and resuspended in PI solution containing 0.1% Triton X-100. Stained nuclei in the sub-G1 region were considered to represent apoptotic cells [65]. For measurement of Annexin V positive cells 5×10^5^ cells were stained with Annexin-V-FITC (Alexis Biochemicals) in Annexin Binding Buffer. Viability of cells was determined with the AlamarBlue assay (AbD Serotec, UK) in a Chameleon MicroplateReader (Hidex, Turku, Finnland) according to manufacturer's instructions.

### Immunoblotting

5×10^6^ cells were lysed on ice in lysis-buffer (50 mM TRIS/HCl, 1 mM EDTA, 150 mM NaCl, 1% IGEPAL, 0.25% Deoxycholic acid sodium salt) with protease inhibitor (complete Mini, Roche Diagnostics, Mannheim, Germany) and protease and phosphatase inhibitors. For LC3 conversion E64d and pepstatin (10 μg/ml each) were added. Protein concentration was measured using Bradford-Reagent (BioRad Laboratories, Munich, Germany). Equal amounts of total protein (20-50 μg/lane) were separated by SDS-PAGE and transferred to nitrocellulose membranes (Schleicher & Schuell, Germany) by a semi-dry blotting device (Hoefer TE70, Amersham Biosciences). Membranes were blocked with PBS blocking buffer containing 0.1% Tween20 and 5% nonfat dry milk or 5% bovine serum albumin, incubated with primary antibodies specific for human PKB, pPKB-Ser473, BCLXL, BECN1, FOXO3, JNK, pJNK-Th183/Tyr185, PARP, CASP3 (Cell Signaling Technology Inc., Boston, USA), BIM, MCL1 (BD Biosciences, Pharmingen, Heidelberg, D), NOXA, pFOXO3-Thr32 (Abcam, Cambridge, UK), LC3, GAPDH (Acris, Herford, Germany), Survivin (R&D System Abingdon, UK), and α-Tubulin (Oncogene Research Products, USA), then washed and incubated with anti-mouse or anti-rabbit horseradish-peroxidase-conjugated secondary antibodies (GE Healthcare, USA). The immunoblots were developed by enhanced chemiluminescence (GE Healthcare, USA) according to the manufacturer's instructions and analyzed in an AutoChemi detection system (UVP, Cambridge, UK). Densitometric analyses were performed using LabWorks software (UVP, Cambridge, UK).

### Co-immunoprecipitation

1×10^7^ cells were lysed in 1% IGEPAL buffer containing protease and phosphatase inhibitors. For each precipitation 1 μg precipitation antibody BECN1 or BCLXL, (Cell Signaling Technology Inc., Boston, USA) or normal IgG control were covalently coupled to Affi-Prep Protein A Support (BioRad Laboratories, Munich, Germany) using dimethylpimelidate dihydrochloride/Borax buffer as described before [44]. Antibody-bead complexes were added to at least 1000 μg lysate and incubated at 4 °C for 6 hours. Protein A-immunocomplexes were washed four times in PBS/IGEPAL-buffer, resuspended in SDS-sample buffer and subjected to SDS-PAGE and blotting. Equal amounts of total protein and cleared supernatants were loaded as controls.

### Live-cell fluorescence microscopy

For analyses of morphological changes, LC3 accumulation, or FOXO3 translocation SH-EP/Ctr, SH-EP/YFP-LC3, SH-EP/shFOXO3-16-cl13-YFP-LC3, NB1/YFP-LC3, NB8/YFP-LC3, NB15/YFP-LC3, or SH-EP/ECFP-FOXO3 cells were seeded on collagen-coated (0.1 mg/ml) glass slides. Mitochondrial staining was performed by MitoTrackerRed/CMXRos (300 nM, Invitrogen, Carlsbad, USA). Nuclei were stained with Hoechst33342 (100 ng/ml, Cambex Bio Sience Walkersville, AZ, USA). For determination of cell death at least 100 cells were analyzed for detachment/nuclear fragmentation. Autophagosomes were counted in at least 30 cells in each of three independent experiments and calculated as autophagosomes/cell. For ROS measurements cells were grown on LabTek Chamber SlidesTM (Nalge Nunc International, USA) coated with 0.1 mg/ml collagen and incubated with MitoTrackerRed/CM-H2XROS (Invitrogen, Carlsbad, CA, USA) according to the manufacturer's instructions (final concentration 500 nM). Tubulin networks were visualized by transfecting SH-EP cells with 2μg pac-GFP1-Tubulin (kind gift of G.Spoden, Innsbruck, Austria) using jetPrime reagent according to the manufacturer's instructions (PeqLab, Erlangen, Germany).

Images were collected at an Axiovert200M microscope equipped with a 20x and a 63x objective (Zeiss, Vienna, Austria). Fluorescence intensity was quantified using Axiovision Software (Zeiss, Vienna, Austria) and relative ROS levels were expressed as % of untreated controls.

### Statistics

Statistical significance of differences between controls and treated cells were calculated using unpaired t-test. All statistical analyses were performed using Graph Pad Prism 4.0 software.

## SUPPLEMENTARY MATERIALS FIGURES



## Abbreviations

MG-2477: 3-cyclopropylmethyl-7-phenyl-3H-pyrrolo[3,2-f]quiolin-9(6H)-one)
BafA: Bafilomycin A1
4OHT: 4-Hydroxytamoxifen
3MA: 3-Methyladenine
NAC: N-acetyl-L-cysteine
RA: all-trans retinoic acid
NGF: nerve growth factor

## References

[1] Brodeur GM, Pritchard J, Berthold F, Carlsen NL, Castel V, Castelberry RP, De Bernardi B, Evans AE, Favrot M, Hedborg F (1993). Revisions of the international criteria for neuroblastoma diagnosis, staging, and response to treatment. J Clin Oncol.

[2] Brodeur GM, Seeger RC, Schwab M, Varmus HE, Bishop JM (1984). Amplification of N-myc in untreated human neuroblastomas correlates with advanced disease stage. Science.

[3] Islam A, Kageyama H, Takada N, Kawamoto T, Takayasu H, Isogai E, Ohira M, Hashizume K, Kobayashi H, Kaneko Y, Nakagawara A (2000). High expression of Survivin, mapped to 17q25, is significantly associated with poor prognostic factors and promotes cell survival in human neuroblastoma. Oncogene.

[4] Opel D, Poremba C, Simon T, Debatin KM, Fulda S (2007). Activation of Akt predicts poor outcome in neuroblastoma. Cancer Res.

[5] Hagenbuchner J, Kuznetsov AV, Obexer P, Ausserlechner MJ (2013). BIRC5/Survivin enhances aerobic glycolysis and drug resistance by altered regulation of the mitochondrial fusion/fission machinery. Oncogene.

[6] Hagenbuchner J, Ausserlechner MJ (2013). Mitochondria and FOXO3: Breath or Die. Frontiers in Physiology.

[7] Dumontet C, Jordan MA (2010). Microtubule-binding agents: a dynamic field of cancer therapeutics. Nat Rev Drug Discov.

[8] Jordan MA, Wilson L (2004). Microtubules as a target for anticancer drugs. Nat Rev Cancer.

[9] Janssen A, Medema RH (2011). Mitosis as an anti-cancer target. Oncogene.

[10] Karna P, Zughaier S, Pannu V, Simmons R, Narayan S, Aneja R (2010). Induction of Reactive Oxygen Species-mediated Autophagy by a Novel Microtubule-modulating Agent. Journal of Biological Chemistry.

[11] Viola G, Bortolozzi R, Hamel E, Moro S, Brun P, Castagliuolo I, Ferlin MG, Basso G (2012). MG-2477, a new tubulin inhibitor, induces autophagy through inhibition of the Akt/mTOR pathway and delayed apoptosis in A549 cells. Biochemical Pharmacology.

[12] Delbridge ARD, Valente LJ, Strasser A (2012). The Role of the Apoptotic Machinery in Tumor Suppression. Cold Spring Harbor Perspectives in Biology.

[13] Galluzzi L, Vitale I, Abrams JM, Alnemri ES, Baehrecke EH, Blagosklonny MV, Dawson TM, Dawson VL, el Deiry WS, Fulda S, Gottlieb E, Green DR, Hengartner MO (2012). Molecular definitions of cell death subroutines: recommendations of the Nomenclature Committee on Cell Death 2012. Cell Death Differ.

[14] Luo S, Rubinsztein DC (2009). Apoptosis blocks Beclin 1-dependent autophagosome synthesis: an effect rescued by Bcl-xL. Cell Death Differ.

[15] Delbridge AR, Strasser A (2015). The BCL-2 protein family, BH3-mimetics and cancer therapy. Cell Death Differ.

[16] Luo S, Rubinsztein DC (2013). BCL2L11/BIM: a novel molecular link between autophagy and apoptosis. Autophagy.

[17] Maiuri MC, Criollo A, Tasdemir E, Vicencio JM, Tajeddine N, Hickman JA, Geneste O, Kroemer G (2007). BH3-only proteins and BH3 mimetics induce autophagy by competitively disrupting the interaction between Beclin 1 and Bcl-2/Bcl-X(L). Autophagy.

[18] Shen HM, Codogno P (2011). Autophagic cell death: Loch Ness monster or endangered species?. Autophagy.

[19] Boya P, Gonzalez-Polo RA, Casares N, Perfettini JL, Dessen P, Larochette N, Metivier D, Meley D, Souquere S, Yoshimori T, Pierron G, Codogno P, Kroemer G (2005). Inhibition of macroautophagy triggers apoptosis. Mol Cell Biol.

[20] Oral O, Oz-Arslan D, Itah Z, Naghavi A, Deveci R, Karacali S, Gozuacik D (2012). Cleavage of Atg3 protein by caspase-8 regulates autophagy during receptor-activated cell death. Apoptosis.

[21] Pagliarini V, Wirawan E, Romagnoli A, Ciccosanti F, Lisi G, Lippens S, Cecconi F, Fimia GM, Vandenabeele P, Corazzari M, Piacentini M (2012). Proteolysis of Ambra1 during apoptosis has a role in the inhibition of the autophagic pro-survival response. Cell Death Differ.

[22] Wirawan E, Vande Walle L, Kersse K, Cornelis S, Claerhout S, Vanoverberghe I, Roelandt R, De Rycke R, Verspurten J, Declercq W, Agostinis P, Vanden Berghe T, Lippens S (2010). Caspase-mediated cleavage of Beclin-1 inactivates Beclin-1-induced autophagy and enhances apoptosis by promoting the release of proapoptotic factors from mitochondria. Cell Death and Dis.

[23] Shimizu S, Kanaseki T, Mizushima N, Mizuta T, Arakawa-Kobayashi S, Thompson CB, Tsujimoto Y (2004). Role of Bcl-2 family proteins in a non-apoptotic programmed cell death dependent on autophagy genes. Nat Cell Biol.

[24] Fazi B, Bursch W, Fimia GM, Nardacci R, Piacentini M, Di Sano F, Piredda L (2008). Fenretinide induces autophagic cell death in caspase-defective breast cancer cells. Autophagy.

[25] Nezis IP, Shravage BV, Sagona AP, Lamark T, Bjorkoy G, Johansen T, Rusten TE, Brech A, Baehrecke EH, Stenmark H (2010). Autophagic degradation of dBruce controls DNA fragmentation in nurse cells during late Drosophila melanogaster oogenesis. J Cell Biol.

[26] Yu L, Wan F, Dutta S, Welsh S, Liu Z, Freundt E, Baehrecke EH, Lenardo M (2006). Autophagic programmed cell death by selective catalase degradation.

[27] Gasparotto V, Castagliuolo I, Ferlin MG (2007). 3-Substituted 7-Phenyl-Pyrroloquinolinones Show Potent Cytotoxic Activity in Human Cancer Cell Lines. Journal of Medicinal Chemistry.

[28] Asnaghi L, Calastretti A, Bevilacqua A, D’Agnano I, Gatti G, Canti G, Delia D, Capaccioli S, Nicolin A (2004). Bcl-2 phosphorylation and apoptosis activated by damaged microtubules require mTOR and are regulated by Akt. Oncogene.

[29] Fujiwara Y, Hosokawa Y, Watanabe K, Tanimura S, Ozaki Ki, Kohno M (2007). Blockade of the phosphatidylinositol-3-kinase –Akt signaling pathway enhances the induction of apoptosis by microtubule-destabilizing agents in tumor cells in which the pathway is constitutively activated. Molecular Cancer Therapeutics.

[30] VanderWeele DJ, Zhou R, Rudin CM (2004). Akt up-regulation increases resistance to microtubule-directed chemotherapeutic agents through mammalian target of rapamycin. Molecular Cancer Therapeutics.

[31] Stock C, Bozsaky E, Watzinger F, Poetschger U, Orel L, Lion T, Kowalska A, Ambros PF (2008). Genes Proximal and Distal to MYCN Are Highly Expressed in Human Neuroblastoma as Visualized by Comparative Expressed Sequence Hybridization. The American Journal of Pathology.

[32] Greene LA, Tischler AS (1976). Establishment of a noradrenergic clonal line of rat adrenal pheochromocytoma cells which respond to nerve growth factor.

[33] Sierra-Fonseca J, Najera O, Martinez-Jurado J, Walker E, Varela-Ramirez A, Khan A, Miranda M, Lamango N, Roychowdhury S (2014). Nerve growth factor induces neurite outgrowth of PC12 cells by promoting Gbetagamma-microtubule interaction. BMC Neuroscience.

[34] Eum KH, Lee M (2011). Crosstalk between autophagy and apoptosis in the regulation of paclitaxel-induced cell death in v-Ha-ras-transformed fibroblasts. Mol Cell Biochem.

[35] Sheen JH, Zoncu R, Kim D, Sabatini DM (2011). Defective regulation of autophagy upon leucine deprivation reveals a targetable liability of human melanoma cells *in vitro* and *in vivo*. Cancer Cell.

[36] Erlich S, Mizrachy L, Segev O, Lindenboim L, Zmira O, Adi-Harel S, Hirsch JA, Stein R, Pinkas-Kramarski R (2007). Differential Interactions Between Beclin 1 and Bcl-2 Family Members. Autophagy.

[37] Niu TK, Cheng Y, Ren X, Yang JM (2010). Interaction of Beclin 1 with survivin regulates sensitivity of human glioma cells to TRAIL-induced apoptosis. FEBS Letters.

[38] Hagenbuchner J, Kiechl-Kohlendorfer U, Obexer P, Ausserlechner MJ (2016). BIRC5/Survivin as a target for glycolysis inhibition in high-stage neuroblastoma. Oncogene.

[39] Haschka MD, Soratroi C, Kirschnek S, Hacker G, Hilbe R, Geley S, Villunger A, Fava LL (2015). The NOXA-MCL1-BIM axis defines lifespan on extended mitotic arrest. Nat Commun.

[40] Hagenbuchner J, Ausserlechner MJ, Porto V, David R, Meister B, Bodner M, Villunger A, Geiger K, Obexer P (2010). The Anti-apoptotic Protein BCL2L1/Bcl-xL Is Neutralized by Pro-apoptotic PMAIP1/Noxa in Neuroblastoma, thereby Determining Bortezomib Sensitivity Independent of Prosurvival MCL1 Expression. Journal of Biological Chemistry.

[41] Tang Y, Hamed HA, Cruickshanks N, Fisher PB, Grant S, Dent P (2012). Obatoclax and Lapatinib Interact to Induce Toxic Autophagy through NOXA. Molecular Pharmacology.

[42] Saita S, Shirane M, Nakayama KI (2013). Selective escape of proteins from the mitochondria during mitophagy. Nat Commun.

[43] Hagenbuchner J, Kuznetsov A, Hermann M, Hausott B, Obexer P, Ausserlechner MJ (2012). FOXO3-induced reactive oxygen species are regulated by BCL2L11 (Bim) and SESN3. Journal of Cell Science.

[44] Hagenbuchner J, Kiechl-Kohlendorfer U, Obexer P, Ausserlechner MJ (2013). A novel Mcl1 variant inhibits apoptosis via increased Bim sequestration. Oncotarget.

[45] Farrell O, Rusten TE, Stenmark H (2013). Phosphoinositide 3-kinases as accelerators and brakes of autophagy. FEBS J.

[46] Obexer P, Geiger K, Ambros PF, Meister B, Ausserlechner MJ (2007). FKHRL1-mediated expression of Noxa and Bim induces apoptosis via the mitochondria in neuroblastoma cells. Cell Death Differ.

[47] Wong CH, Iskandar KB, Yadav SK, Hirpara JL, Loh T, Pervaiz S (2010). Simultaneous Induction of Non-Canonical Autophagy and Apoptosis in Cancer Cells by ROS-Dependent ERK and JNK Activation. PLoS One.

[48] Liu YL, Lai F, Wilmott JS, Guang Yan X, Liu XY, Luan Q, Tang Guo S, Jiang CC, Tseng HY, Scolyer RA, Jin L, Zhang XD (2014). Noxa upregulation by oncogenic activation of MEK/ERK through CREB promotes autophagy in human melanoma cells. Oncotarget.

[49] Mammucari C, Milan G, Romanello V, Masiero E, Rudolf R, Del Piccolo P, Burden SJ, Di Lisi R, Sandri C, Zhao J, Goldberg AL, Schiaffino S, Sandri M (2007). FoxO3 Controls Autophagy in Skeletal Muscle *In Vivo*. Cell Metabolism.

[50] Essers MA, Weijzen S, Vries-Smits AM, Saarloos I, de Ruiter ND, Bos JL, Burgering BM (2004). FOXO transcription factor activation by oxidative stress mediated by the small GTPase Ral and JNK. EMBO J.

[51] Ausserlechner MJ, Hagenbuchner J (2016). Mitochondrial survivin - an Achilles’ heel in cancer chemoresistance. Molecular & Cellular Oncology.

[52] Obexer P, Hagenbuchner J, Unterkircher T, Sachsenmaier N, Seifarth C, Bock G, Porto V, Geiger K, Ausserlechner M (2009). Repression of BIRC5/survivin by FOXO3/FKHRL1 sensitizes human neuroblastoma cells to DNA damage-induced apoptosis. Mol Biol Cell.

[53] Tang TT, Dowbenko D, Jackson A, Toney L, Lewin DA, Dent AL, Lasky LA (2002). The forkhead transcription factor AFX activates apoptosis by induction of the BCL-6 transcriptional repressor. J Biol Chem.

[54] Sartelet H, Rougemont AL, Fabre M, Castaing M, Duval M, Fetni R, Michiels S, Beaunoyer M, Vassal G (2011). Activation of the phosphatidylinositol 3’-kinase/AKT pathway in neuroblastoma and its regulation by thioredoxin 1. Human Pathology.

[55] Kyrylenko S, Roschier M, Korhonen P, Salminen A (1999). Regulation of PTEN expression in neuronal apoptosis. Molecular Brain Research.

[56] Thiele C.J, Masters J.R.W (1998). Neuroblastoma Cell Lines, in: “Human Cell Culture”.

[57] Storlazzi CT, Lonoce A, Guastadisegni MC, Trombetta D, D’Addabbo P, Daniele G, L’Abbate A, Macchia G, Surace C, Kok K, Ullmann R, Purgato S, Palumbo O (2010). Gene amplification as double minutes or homogeneously staining regions in solid tumors: origin and structure. Genome Res.

[58] Ushmorov A, Debatin KM, Beltinger C (2005). Growth inhibition of murine neuroblastoma cells by C-Myc with cell cycle arrest in G2/M. Cancer Biology & Therapy.

[59] Luo S, Garcia-Arencibia M, Zhao R, Puri C, Toh P, Sadiq O, Rubinsztein D (2012). Bim Inhibits Autophagy by Recruiting Beclin 1 to Microtubules. Molecular Cell.

[60] Aveic S, Pantile M, Seydel A, Esposito MR, Zanon C, Li G, Tonini GP (2016). Combating autophagy is a strategy to increase cytotoxic effects of novel ALK inhibitor entrectinib in neuroblastoma cells. Oncotarget.

[61] Kim AD, Kang KA, Kim HS, Kim DH, Choi YH, Lee SJ, Kim HS, Hyun JW (2013). A ginseng metabolite, compound K, induces autophagy and apoptosis via generation of reactive oxygen species and activation of JNK in human colon cancer cells. Cell Death Dis.

[62] Ling LU, Tan KB, Lin H, Chiu GNC (2011). The role of reactive oxygen species and autophagy in safingol-induced cell death. Cell Death and Dis.

[63] Xie CM, Chan WY, Yu S, Zhao J, Cheng CHK (2011). Bufalin induces autophagy-mediated cell death in human colon cancer cells through reactive oxygen species generation and JNK activation. Free Radical Biology and Medicine.

[64] Salcher S, Hagenbuchner J, Geiger K, Seiter MA, Rainer J, Kofler R, Hermann M, Kiechl-Kohlendorfer U, Ausserlechner MJ, Obexer P (2014). C10ORF10/DEPP, a transcriptional target of FOXO3, regulates ROS-sensitivity in human neuroblastoma. Mol Cancer.

[65] Nicoletti I, Migliorati G, Pagliacci MC, Grignani F, Riccardi C (1991). A rapid and simple method for measuring thymocyte apoptosis by propidium iodide staining and flow cytometry. J Immunol Methods.

