# Supplementary material for: The tubulin inhibitor MG-2477 induces autophagy-regulated cell death, ROS accumulation and activation of FOXO3 in neuroblastoma
Source: Oncotarget. 2017 Mar 22;8(19):32009–26. doi: 10.18632/oncotarget.16434 (PMC5458265; doi:10.18632/oncotarget.16434)
Supplement: Supplementary file 1 [file oncotarget-08-32009-s001.pdf]

The tubulin inhibitor MG-2477 induces autophagy-regulated cell death, ROS accumulation and activation of FOXO3 in neuroblastoma

SUPPLEMENTARY MATERIALS

SUPPLEMENTARY FIGURES

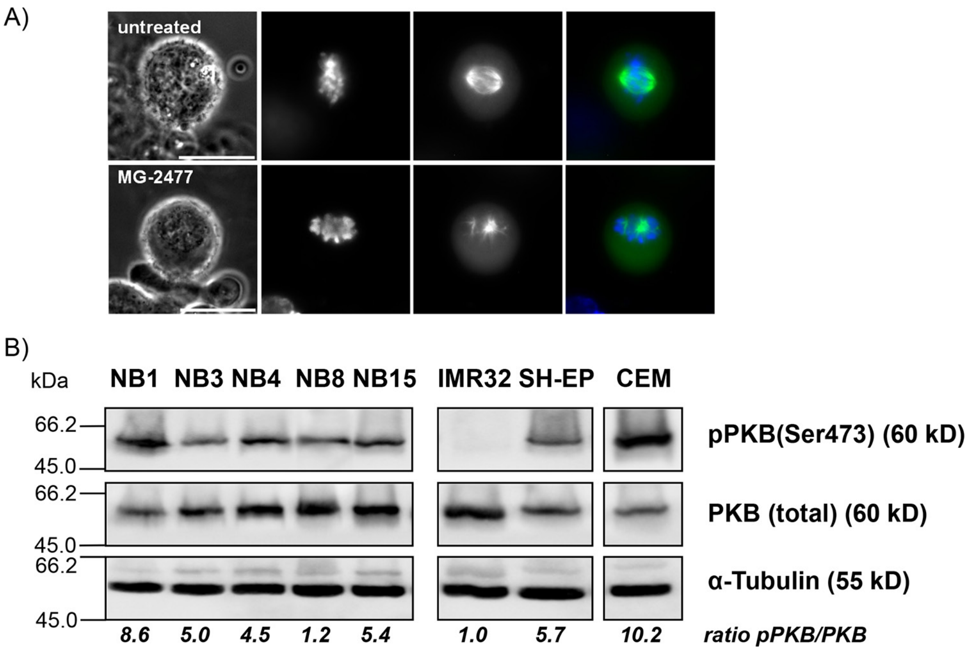

Supplementary Figure 1: (A) Live-cell images of SH-EP cells transfected with pac-GFP1-Tubulin after treatment with 50 nM MG-2477 for six hours. Nuclei/DNA were visualized by Hoechst33342 staining (100 ng/ml). Bar represents 10 µm. **(B)** Neuroblastoma cells differ in the pPKB/PKB ratio. Cell lysates of the neuroblastoma cell lines NB1, NB3, NB4, NB8, NB15, IMR32, and SH-EP were subjected to immunoblot and analyzed for pPKB(Ser473) and total PKB levels. α-Tubulin served as loading control. The PTEN-deficient leukemia cell line CEM-C7H2-2c8 served as positive control for pPKB. Ratio between pPKB and PKB was determined by densitometry (IMR32 was set as 1.0).

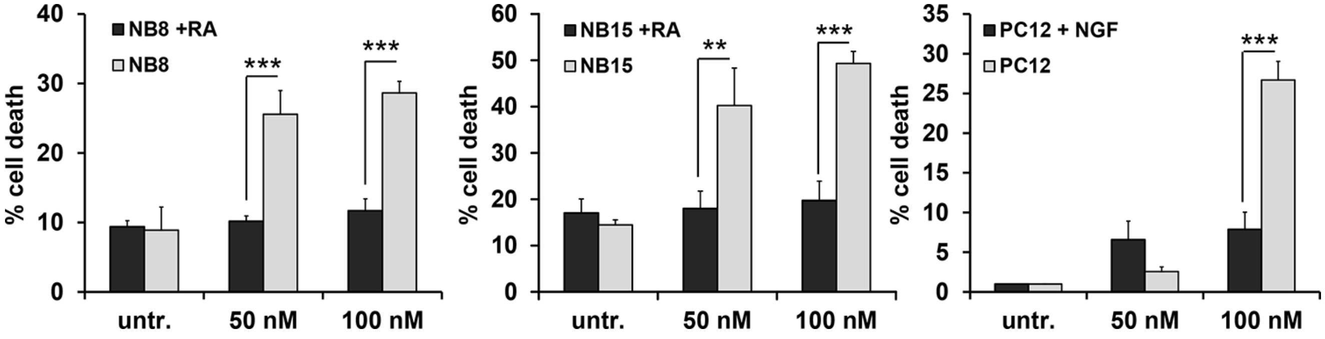

Supplementary Figure 2: Differentiated neuroblastoma cells and pheochromocytoma PC12 cells are insensitive to MG-2477 treatment. NB8 and NB15 cells were differentiated for five days with 15 µM all-trans-retinoic acid (RA) and PC12 cells were differentiated for 72 hours with 100 nM nerve growth factor (NGF). Undifferentiated as well as differentiated cells were treated with increasing doses of MG-2477 for 48 (NB8, NB15 cells) or 24 hours (PC12 cells). Cell death was assessed by PI-FACS analyses. Shown is the mean of three independent experiments. Statistical difference between differentiated and undifferentiated cells was assessed by unpaired t-test (significantly different ** $P<0.01$;***$P<0.001$).

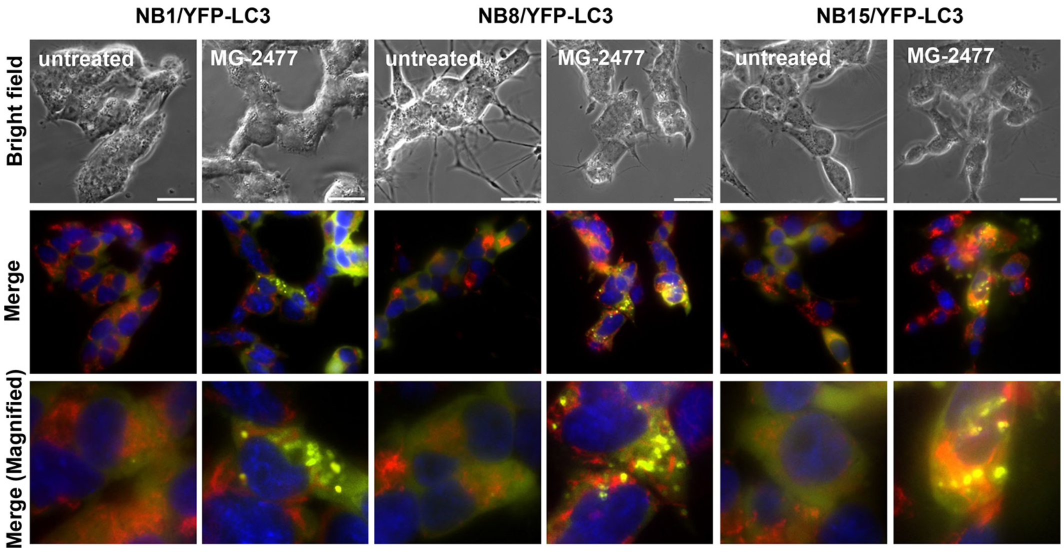

Supplementary Figure 3: Autophagosome formation was monitored via live-cell microscopy in NB1, NB8, and NB15 cells retrovirally infected with a vector coding for YFP-LC3. Cells were treated for one hour with 50 nM MG-2477. Mitochondria were stained with MitoTrackerRed/CMXRos (300 nM), nuclei were stained with Hoechst33342 (100 ng/ml). Bar is 20 µm.

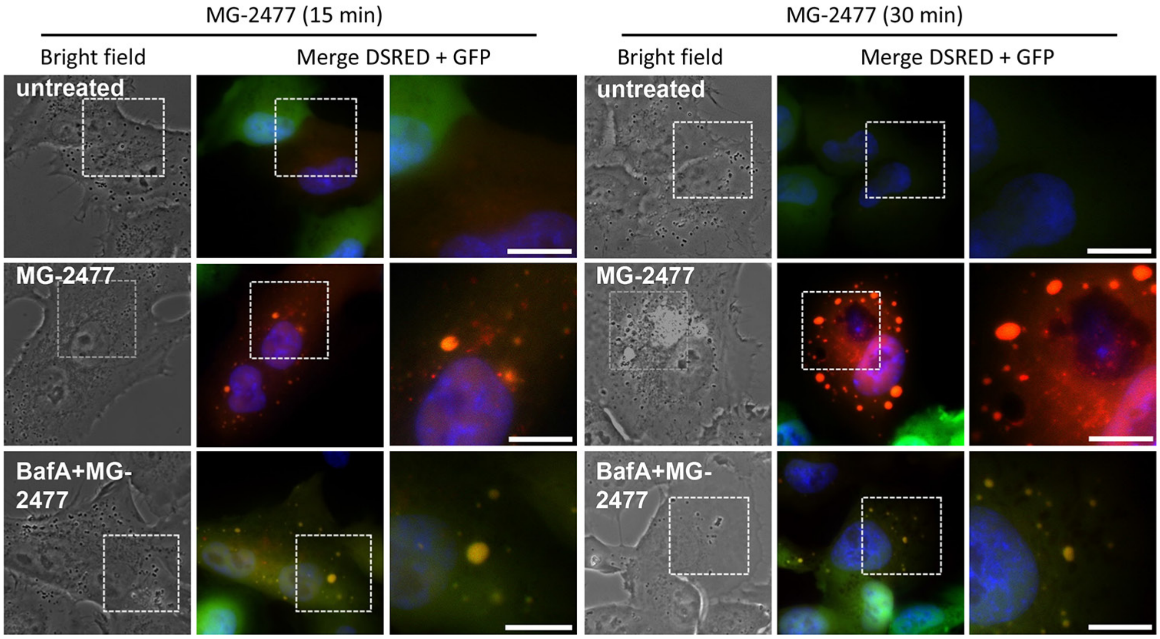

Supplementary Figure 4: MG-2477 induces autophagic flux. SH-EP/dsRed-LC3-GFP cells expressing a dsRED-LC3-GFP protein were treated with MG-2477 for 15 or 30 minutes with or without pre-incubation with 100 nM BafilomycinA (BafA). Autophagic flux was visualized by live cell microscopy of autophagosome and autophagolysosome formation. Nuclei were stained with Hoechst33342 (100 ng/ml). Bar is 10µm.

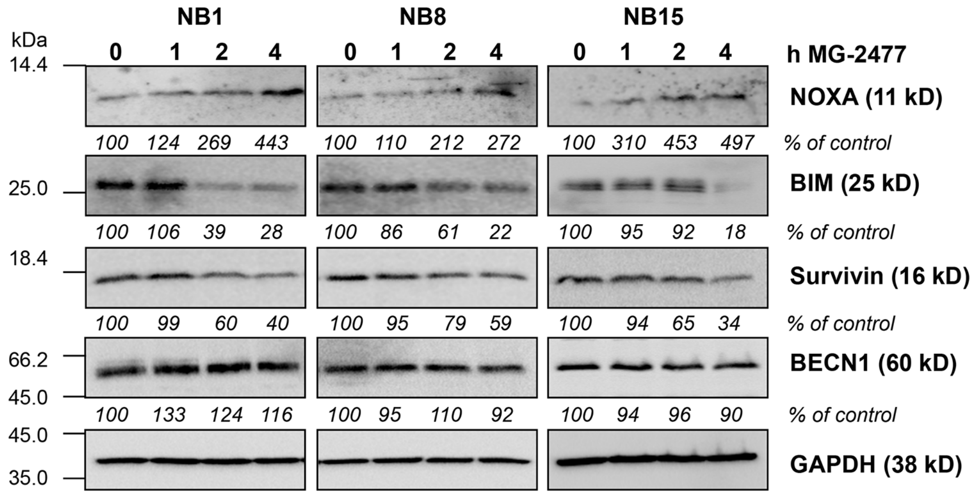

Supplementary Figure 5: Immunoblot analyses of NOXA, BIM, Survivin and BECN1 expression after treatment with MG-2477 for the indicated time. NB1, NB8, and NB15 cells were treated with 50 nM MG-2477. GAPDH served as loading control. Densitometric analyses were performed with Labworks software. Untreated cells were set as 100%.

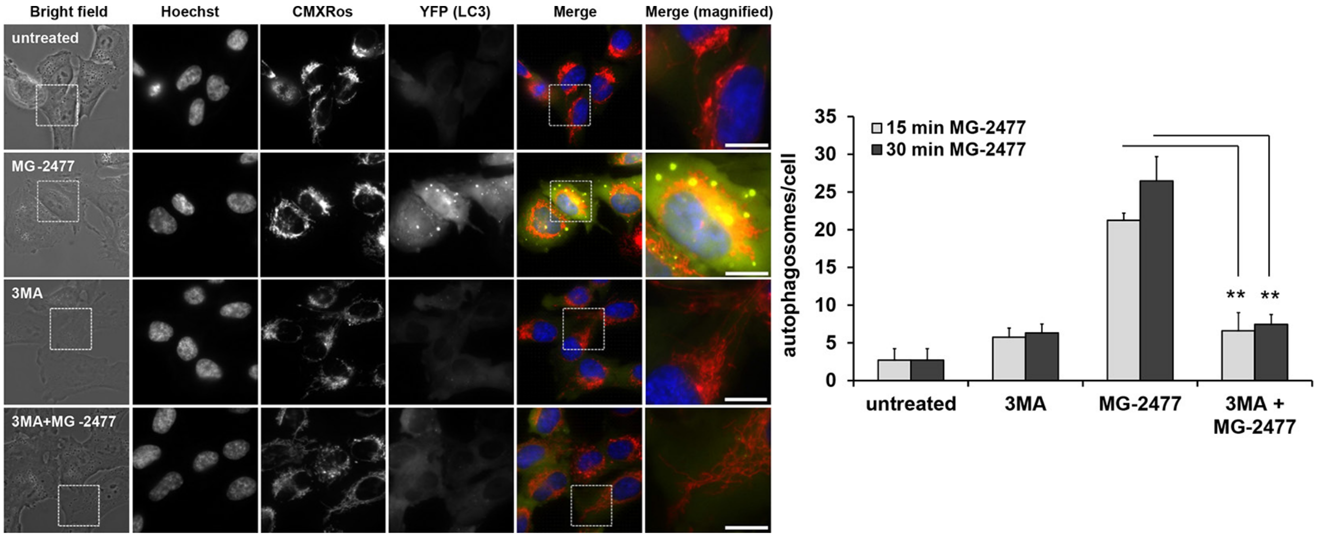

Supplementary Figure 6: 3MA prevents MG-2477 induced formation of autophagosomes. SH-EP/YFP-LC3 cells were pre-incubated with 0.5 mM 3MA for 15 minutes before 50 nM MG-2477 was added for another 15 or 30 minutes. Shown are representative pictures after 15 minutes (left panel) and the mean of three independent experiments (right panel). For each experiment autophagosomes were counted in at least 30 cells (**P<0.01, unpaired t-test).

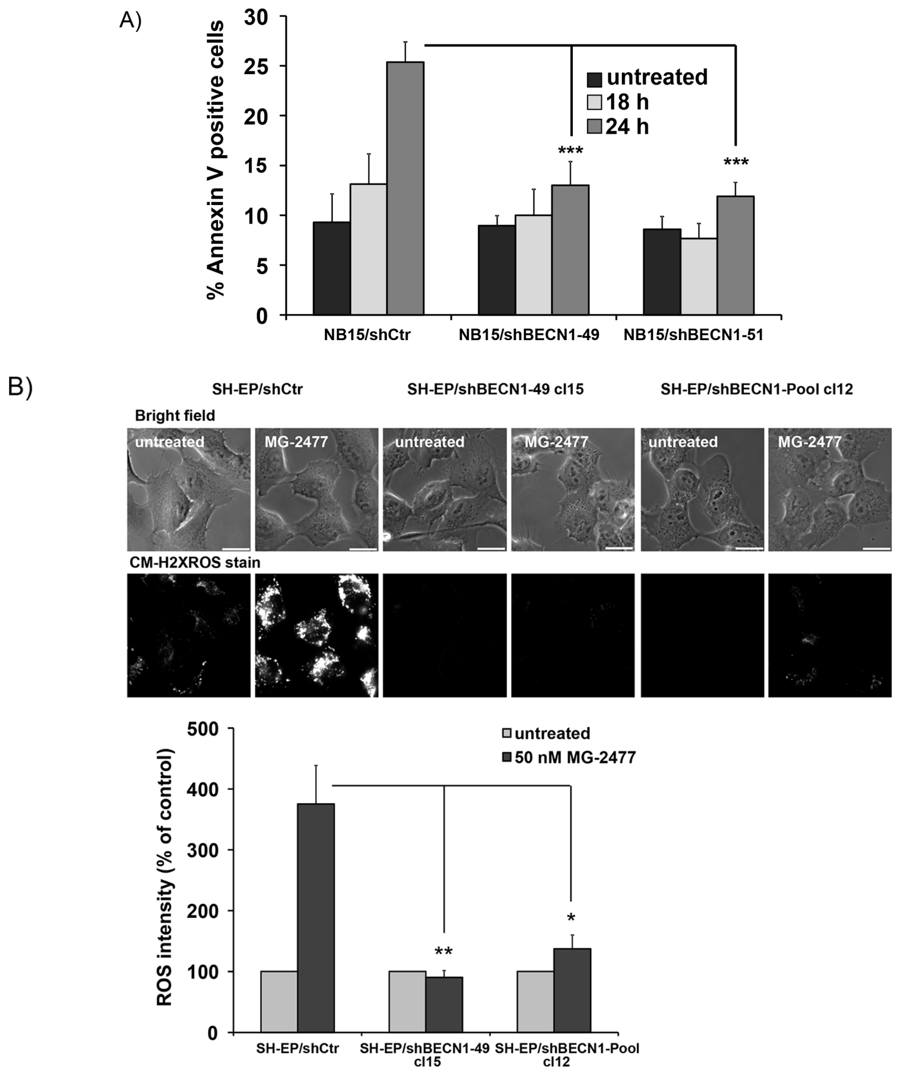

Supplementary Figure 7: (A) NB15/shCtr, NB15/shBECN1-49 and NB15/shBECN1-51 cells were treated for 18 or 24 hours with MG-2477 (50 nM). Cell death was assessed by AnnexinV-FITC staining *via* flow cytometry. Shown is the mean of four independent experiments. ***P<0.001. **(B)** ROS accumulation was measured by CM-H2XROS (500 nM) in SH-EP/shCtr and SH-EP/shBECN1-49 cl15 and SH-EP/shBECN1-Pool cl12 cells after treatment with 50 nM MG-2477 for 30 minutes. Representative micrographs are shown. In each of four independent experiments the fluorescence intensity of more than at least 30 cells was measured by densitometry. * P<0.05; ** P<0.01.

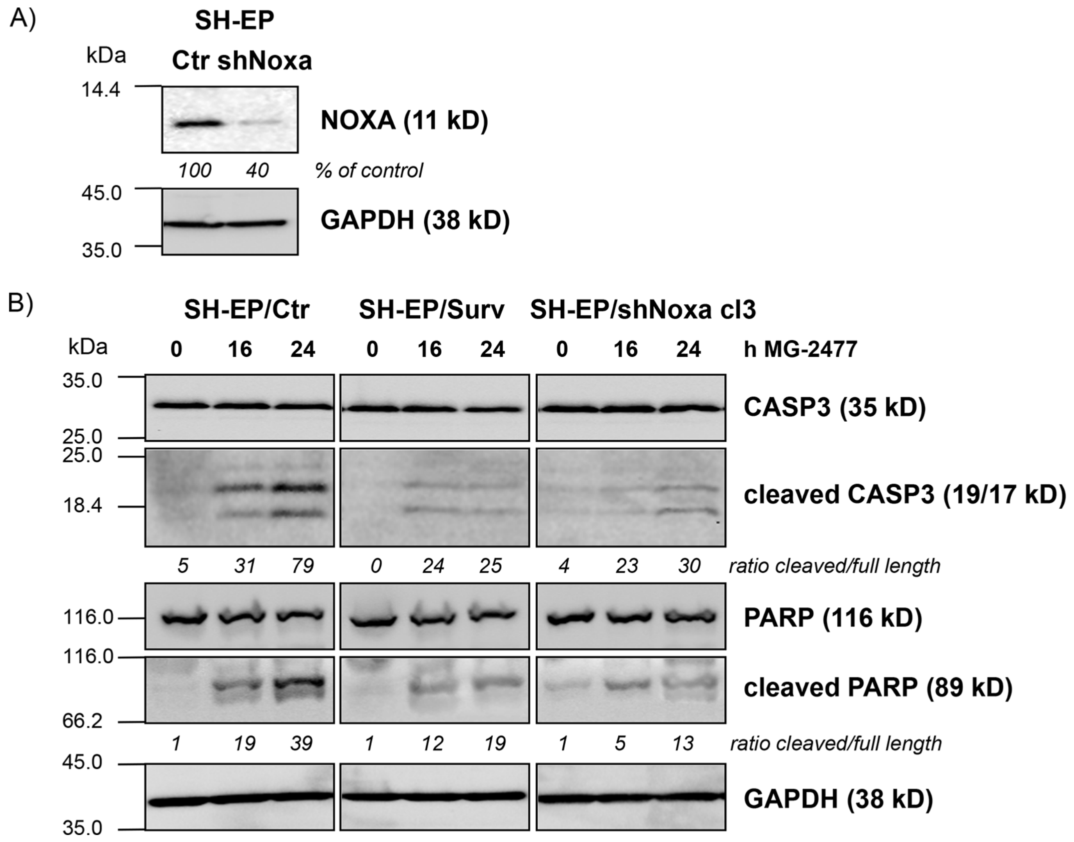

Supplementary Figure 8: (A) Knockdown of NOXA was verified by immunoblot analyses of SHEP/Ctr and SH-EP/shNoxa cl3 cells. GAPDH served as loading control. **(B)** SH-EP/Ctr, SH-EP/Surv and SH-EP/shNoxa cl3 cells were treated with 50 nM MG-2477 for 16 and 24 hours. Cell lysates were subjected to immunoblot analyses for CASP3 and PARP cleavage. GAPDH served as loading control. Densitometric analyses were done using Labworks software and expressed as cleaved/full length ratio.

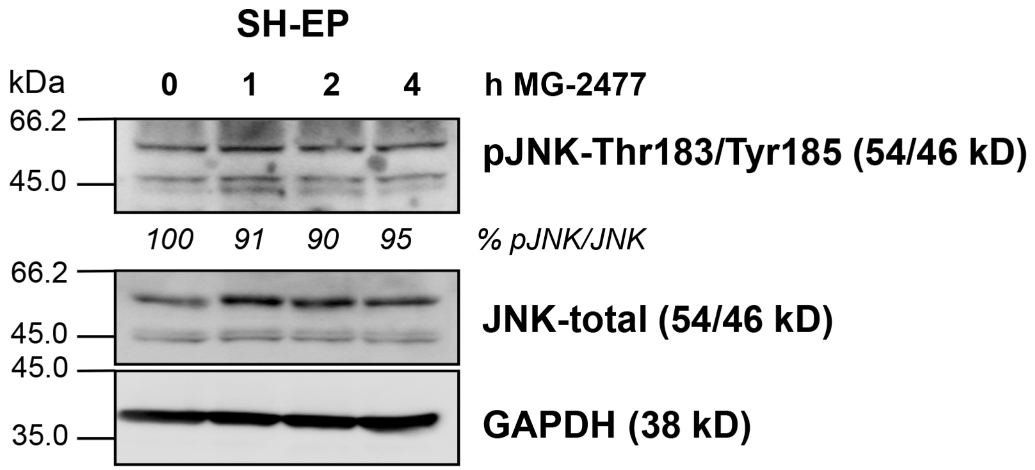

Supplementary Figure 9: JNK is not a critical regulator of MG-2477-induced death. Immunoblot analyses of cell lysates treated with 50 nM MG-2477 for the times indicated. Antibodies were directed against pJNK-Thr183/Tyr185 and total JNK. GAPDH served as loading control. Densitometric analyses were performed using LabWorks software to calculate the pJNK/JNK ratio.

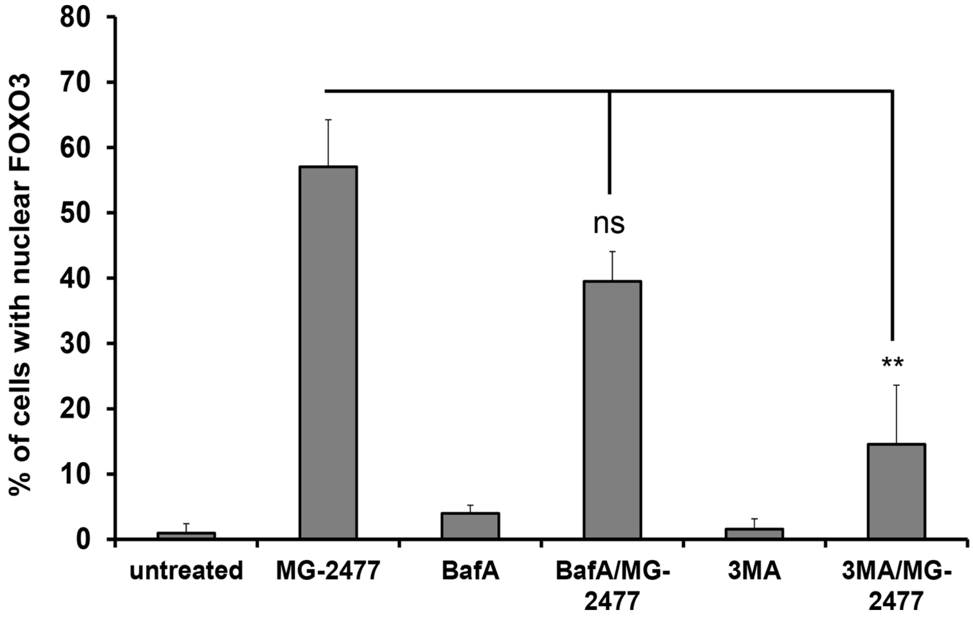

Supplementary Figure 10: MG-2477-treatment causes nuclear accumulation of FOXO3. Statistical analyses of live-cell images of SH-EP/ECFP-FOXO3 cells after treatment with 50 nM MG-2477 for up to 60 minutes alone or in combination with 0.5 mM 3MA or 100 nM BafA (preincubated for 30 minutes). For statistics at least 100 cells from three independent experiments were counted and analyzed for nuclear or cytoplasmic ECFP-FOXO3 localization. Statistical differences were assessed by unpaired t-test (**P<0.01).

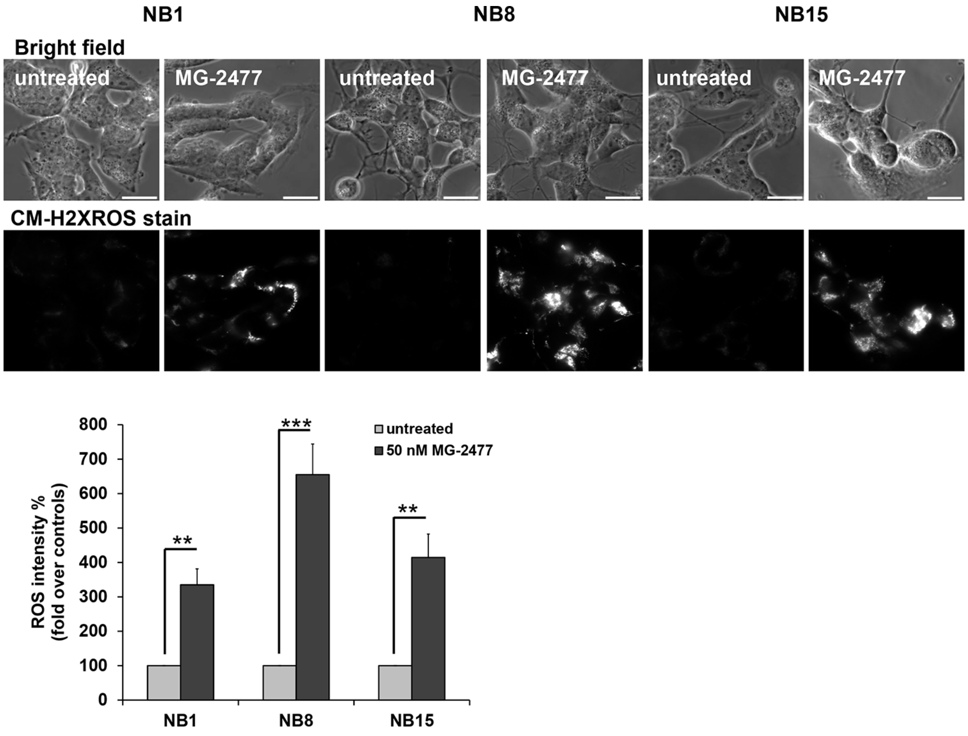

Supplementary Figure 11: ROS accumulation was measured by CM-H2XROS (500 nM) in NB1, NB8, and NB15 cells after treatment with 50 nM MG-2477 for 30 minutes. For the quantification of cellular ROS intensity in each experiment more than 30 cells were quantified by densitometry (four independent experiments). Statistical analyses: student's t-test; **P<0.01; ***P<0.001.

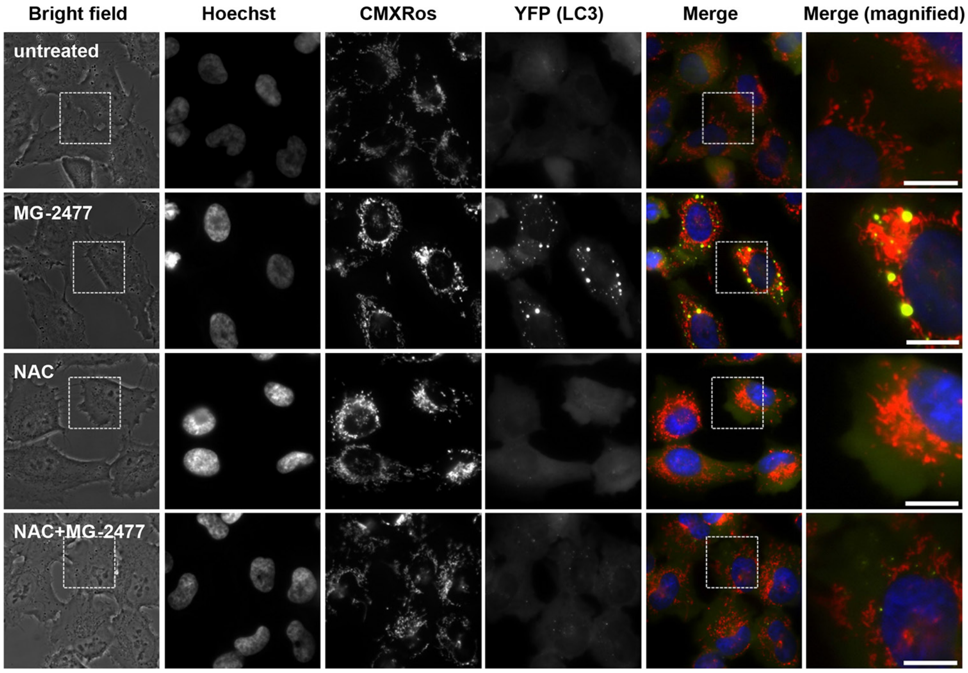

Supplementary Figure 12: NAC reduces MG-2477-induced formation of autophagosomes. SH-EP/YFP-LC3 cells were pre-incubated with 5 mM NAC for 15 minutes before 50 nM MG-2477 was added for another 30 minutes. Shown are representative micrographs.

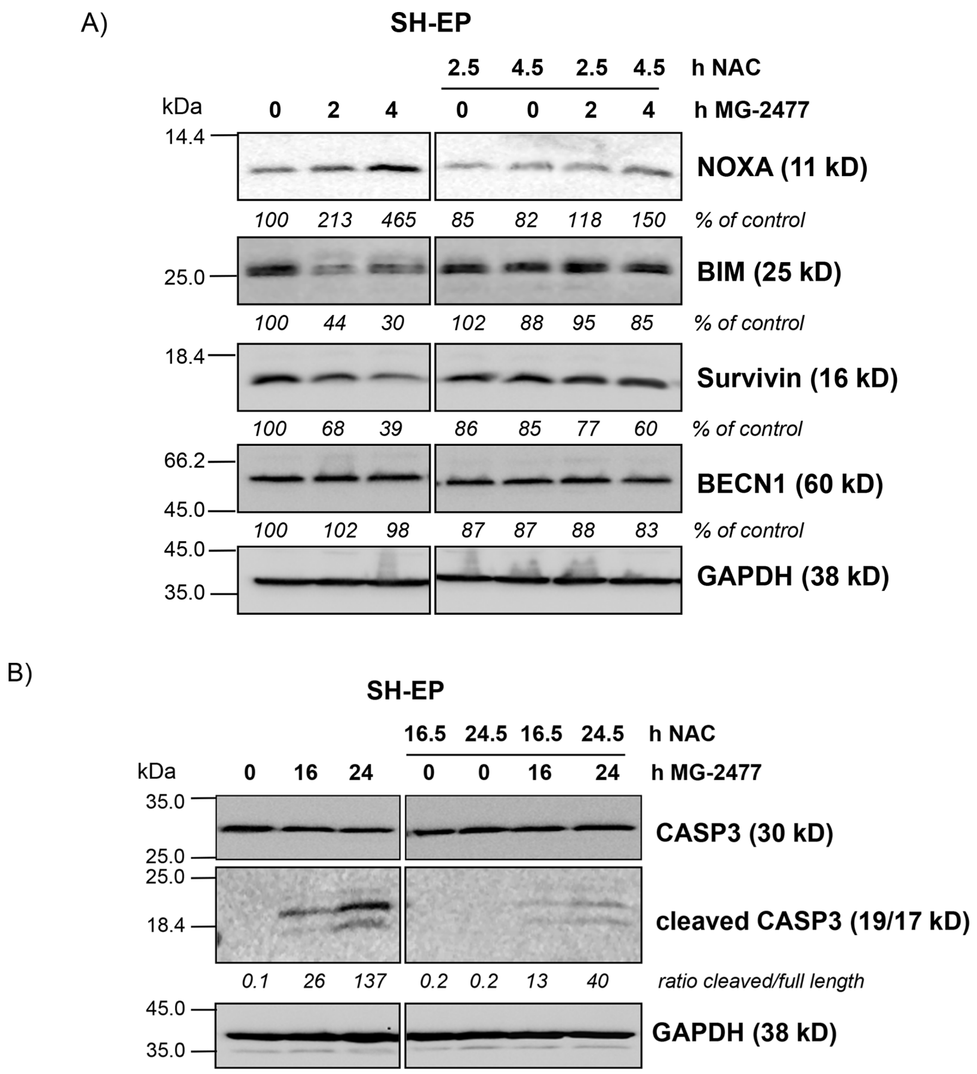

Supplementary Figure 13: (A) SH-EP cells were preincubated with 5 mM NAC for 30 minutes before MG-2477 (50 nM) was added for two or four hours, respectively. NAC-treated controls for two and four hours time points of MG-2477 treatment are shown in lane four (2.5 h NAC) and five (4.5 h NAC), respectively. Cell lysates were subjected to immunoblot analyses of NOXA, BIM, BECN1 and Survivin expression. GAPDH served as loading control. Untreated cells were set as 100% for densitometric analyses. **(B)** SH-EP cells were preincubated with 5 mM NAC for 30 minutes before MG-2477 (50 nM) was added for another 16 or 24 hours. Cell lysates were subjected to immunoblot analyses of CASP3 and PARP. GAPDH served as loading control. Densitometric analyses were done using Labworks software and expressed as cleaved/full length ratio.

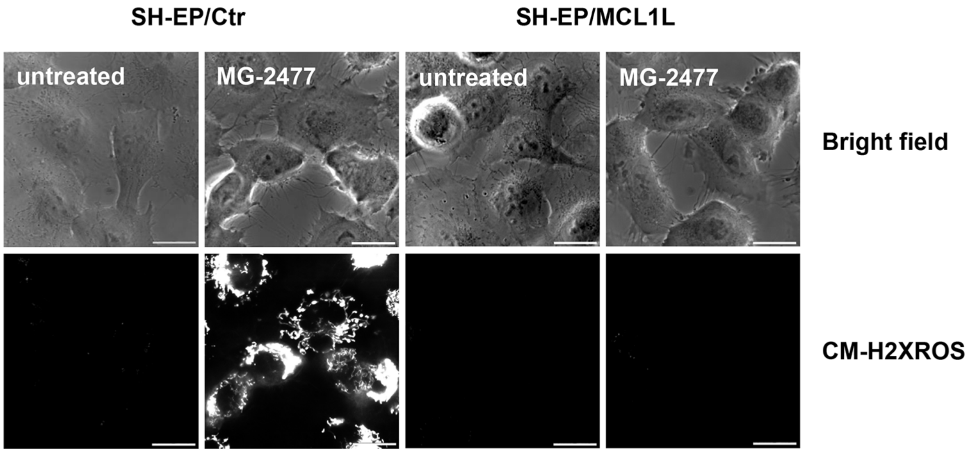

Supplementary Figure 14: MCL1L blocks ROS formation during MG-2477 treatment. SH-EP/Ctr and SH-EP/MCL1L cells were treated with 50 nM MG-2477 for 30 minutes and analyzed for ROS-accumulation with 500 nM CM-H2XROS. Bar is 20 µm.

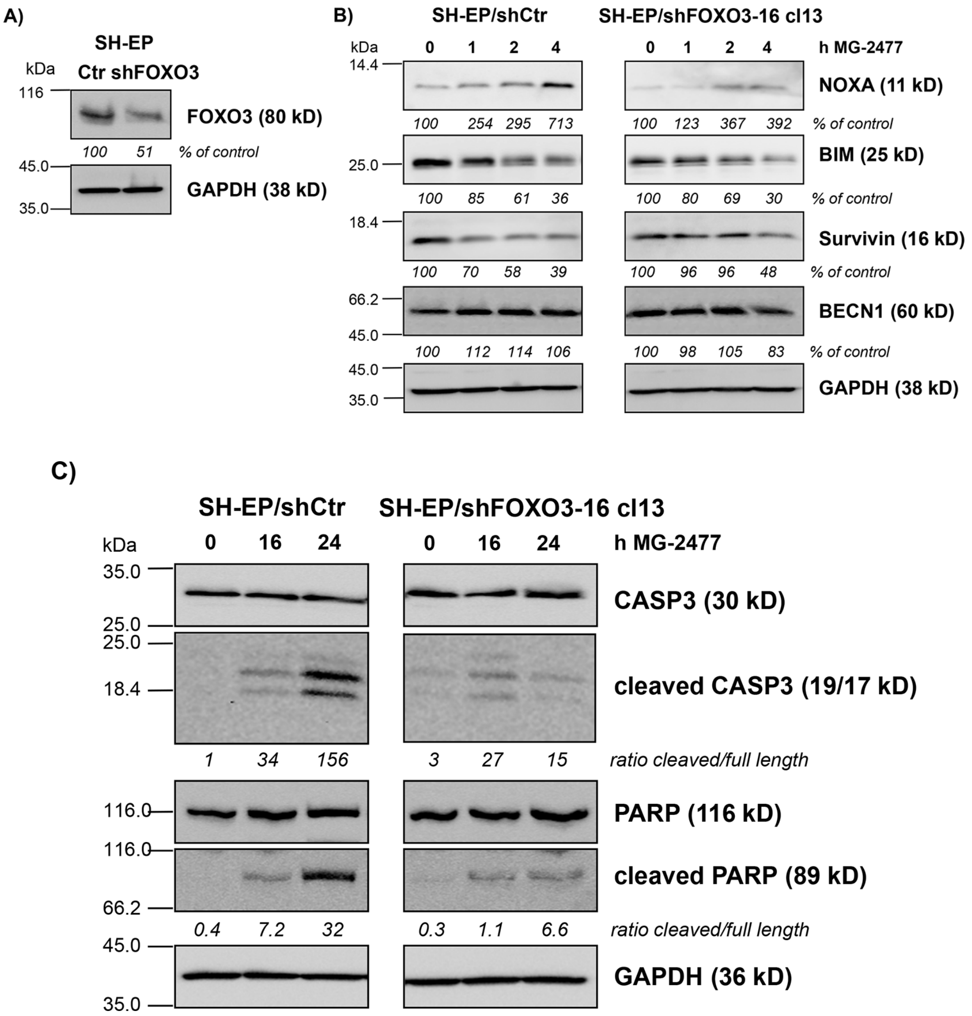

Supplementary Figure 15: (A) Knockdown of FOXO3 was verified by immunoblot analyses of SHEP/shCtr and SH-EP/shFOXO3-16-cl13 cells. GAPDH served as loading control. **(B)** Immunoblot analyses of NOXA, BIM, BECN1, and Survivin expression after treatment of SH-EP/shCtr and SH-EP/shFOXO3-16-cl13 cells with 50 nM MG-2477 for the times indicated. GAPDH served as loading control. Densitometric analyses were performed with Labworks software. Untreated cells were set as 100%. **(C)** SH-EP/shCtr and SH-EP/shFOXO3-16-cl13 cells were treated with 50 nM MG-2477 for 16 and 24 hours. Cell lysates were subjected to immunoblot analyses for CASP3 and PARP cleavage. GAPDH served as loading control. Densitometric analyses were done using Labworks software and expressed as cleaved/full length ratio.

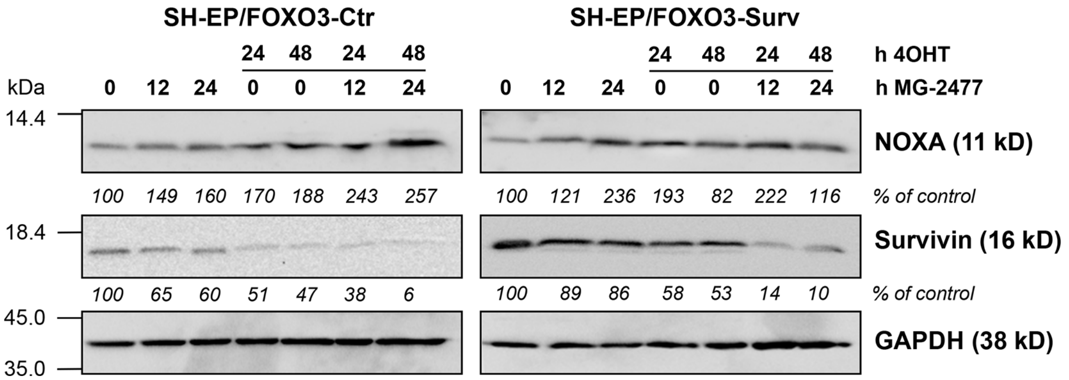

Supplementary Figure 16: SH-EP/FOXO3-Ctr and SH-EP/FOXO3-Surv cells were pre-incubated with 20 nM 4OHT for either 12 or 24 hours before 35 nM MG-2477 were added for additional 12 or 24 hours, respectively. 4OHT treated controls for the 12 and 24 hours time points of MG-2477 treatment are shown in lane 4 (24 h 4OHT) and 5 (48 h 4OHT), respectively. Cell lysates were subjected to immunoblot analyses for NOXA or Survivin expression. GAPDH served as loading control. Densitometry analyses were done using Labworks software. Untreated cells were set as 100%.
